# The Emergence of Alternative 3′ and 5′ Splice Site Exons from Constitutive Exons

**DOI:** 10.1371/journal.pcbi.0030095

**Published:** 2007-05-25

**Authors:** Eli Koren, Galit Lev-Maor, Gil Ast

**Affiliations:** Department of Human Molecular Genetics, Sackler Faculty of Medicine, Tel Aviv University, Tel Aviv, Israel; Universitat Pompeu Fabra, Spain

## Abstract

Alternative 3′ and 5′ splice site (ss) events constitute a significant part of all alternative splicing events. These events were also found to be related to several aberrant splicing diseases. However, only few of the characteristics that distinguish these events from alternative cassette exons are known currently. In this study, we compared the characteristics of constitutive exons, alternative cassette exons, and alternative 3′ss and 5′ss exons. The results revealed that alternative 3′ss and 5′ss exons are an intermediate state between constitutive and alternative cassette exons, where the constitutive side resembles constitutive exons, and the alternative side resembles alternative cassette exons. The results also show that alternative 3′ss and 5′ss exons exhibit low levels of symmetry (frame-preserving), similar to constitutive exons, whereas the sequence between the two alternative splice sites shows high symmetry levels, similar to alternative cassette exons. In addition, flanking intronic conservation analysis revealed that exons whose alternative splice sites are at least nine nucleotides apart show a high conservation level, indicating intronic participation in the regulation of their splicing, whereas exons whose alternative splice sites are fewer than nine nucleotides apart show a low conservation level. Further examination of these exons, spanning seven vertebrate species, suggests an evolutionary model in which the alternative state is a derivative of an ancestral constitutive exon, where a mutation inside the exon or along the flanking intron resulted in the creation of a new splice site that competes with the original one, leading to alternative splice site selection. This model was validated experimentally on four exons, showing that they indeed originated from constitutive exons that acquired a new competing splice site during evolution.

## Author Summary

Alternative splicing is the mechanism that is responsible for the creation of multiple mRNA products from a single gene. It is considered a key player in genomic complexity achievement. Alternative 3′ and 5′ splicing events in which part of the exon is alternatively included or excluded in the mRNA constitute a significant part of all alternative splicing events, and yet little is known regarding their regulation mechanism and the evolutionary background that led to their creation. We show that alternative 3′ and 5′ splice site exons resemble constitutive exons. However, their alternative sequence resembles alternative cassette exons. Comparative genomics spanning seven vertebrate species suggests an evolutionary model in which the alternative state is a derivative of an ancestral constitutive exon, where a mutation inside the exon or along the flanking intron resulted in the creation of a new splice site that competes with the original one, leading to alternative splice site selection. This model was validated experimentally, showing that during evolution mutations shifted constitutive exons to undergo alternative 3′ and 5′ splicing.

## Introduction

The human genome sequencing project has led to the understanding that total gene number is not indicative of a higher level of phenotypic complexity, as the number of human genes is ~25,000, only slightly higher than the nematode (~19,000 genes) and lower than rice (~40,000 genes) [1,2]. The mechanism that was proposed to resolve this discrepancy is alternative splicing, in which several mRNA isoforms are generated from a single gene through the alternative selection of 3′ss and/or 5′ss, producing several functional proteins [3,4]. There are five major forms of alternative splicing: exon skipping (also known as cassette exons), in which the exon as a whole either is included in the mature mRNA transcript or is skipped. Exon skipping is the most common alternative splicing event and accounts for 38% of conserved alternative splicing events between human and mouse. Alternative 3′ss exons (A3Es) and 5′ss exons (A5Es) account for ~18% and ~8% of the human–mouse conserved events, respectively. These exons are flanked on one side by a constitutive splice site (fixated) and on the other side by two (or more) competing alternative splice sites, resulting in an alternative region (extension) that either is included in the transcript or is excluded. Intron retention accounts for fewer than 3% of the human–mouse conserved alternative splicing events, whereas the remaining ~33% are different types of complex events [5,6]. Four splice signals are essential for accurate splicing: 5′ and 3′ splice sites (5′ss and 3′ss), the polypyrimidine tract, and the branch site sequence [7]. However, these signals solely can't support proper splice site selection and proper splicing. *Cis*-acting regulatory elements called exonic splicing enhancers (ESEs) and exonic splicing silencers (ESSs; ESEs + ESSs are also termed ESRs) were found to be involved in the regulation of the alternative splicing process [8]. These elements have the major effect when located in proximity to the alternative splice site [9–12].

Previous studies focused their research mainly on cassette exons in the quest of identifying the regulatory mechanism governing their splicing and the evolutionary background that led to their creation [13,14]. In addition, other studies have tried to examine the biological significance and evolutionary mechanism of intron-retained exons [15,16]. On the other hand, A3Es and A5Es are relatively more poorly characterized, even though they were found to be related to several diseases caused by aberrant splicing generated by mutations [17–19]. Thus, it is important to widen the knowledge regarding their regulation, characteristics, and evolutionary origin. Examination of A3Es and A5Es reveals that their alternative region is highly diverse in terms of length. However, ~50% of A3Es have alternative splice sites that are exactly 3 bp apart (known as the NAGNAG motif). This was one of the reasons that led several studies to examine a subset of A3Es and A5Es (containing a short alternative region, 1–4 bp in length), in an attempt to learn whether they are controlled by a highly complex regulatory mechanism and serve as a fine-tuning mechanism for protein functionality [20–22] or are merely a meter of noise of the splicing machinery [23]. A previous study showed that in A3Es, an additional polypyrimidine tract (PPT) between the two 3′ss appears only when the distance between them is more than 8 bp, whereas in A5Es, the GT dinucleotide located 4 bp downstream of the authentic GT in the 5′ss sequence (GTNNGT) can serve as a competing 5′ss [24]. Other studies have aimed to develop tools that will enable us to identify new A3Es and A5Es based on different criteria [25,26]. However, the evolutionary mechanism that led to their creation is largely unknown.

Comparative genomics has been used as a powerful tool for the identification of functional biological features. The guideline directing this approach is that a high conservation level implies an essential biological function—structural or regulatory [27]. Human–mouse comparison is the most commonly used in research, because it is estimated that the two species diverged 75–130 million years ago and, hence, had enough time to accumulate mutations on the one hand while still maintaining high levels of homology on the other. In addition, nearly all human genes (99%) have a mouse ortholog with a high (88%) protein coding sequence resemblance [28]. Moreover, their genomes have been explored widely and their transcriptome covered by millions of EST sequences, making it easier to perform large-scale analyses.

It has been previously shown that constitutive and alternative cassette exons differ from each other in several features, such as: splice site strength, exon length, conservation level between human and mouse (exon identity), divisibility-by-3 (symmetry), and K_A_/K_S_ ratio test [6,14,29]. These discriminating features were also used to explore the regulatory mechanism that is required to ensure the proper splicing of these exons.

A3Es and A5Es can produce (at least) two splice transcripts: one contains the extension, and the other excludes it. These transcripts can be formed in different ratios, i.e., one can be more abundant (major form) compared with the other (minor form). Thus, the more common form of each exon can allude to its evolutionary background. Thus, we examined each of the aforementioned characteristics in A3Es and A5Es, subdivided into two subgroups according to the common form, and compared them with constitutive and alternative cassette exons to understand the overall regulatory mechanism that directs the proper selection of one alternative splice site over the other. Our analysis was based on a unified dataset of human–mouse orthologous exons comprising constitutive exons, alternative cassette exons, A3Es, and A5Es [6,30,31]. The analysis was expanded further to multiple species alignment to acquire knowledge on the evolutionary background of A3Es and A5Es from both subgroups. Our results suggest an evolutionary model for the creation of new A3Es and A5Es. According to this model, A3Es and A5Es originated from constitutive exons that acquired a new functional splice site as a result of a mutation in the exon or along the intron. This splice site started to compete with the authentic splice site, leading to alternative splice site selection. We provide computational and experimental evidence supporting this hypothesis.

## Results

### Characterization of Alternative 3′ and 5′ Exons

To identify unique characteristics of A3Es and A5Es, we examined several features that can differentiate these exons. We compiled four datasets of human–mouse orthologous exons, including their flanking introns: 45,553 constitutively spliced exons, 757 alternative cassette exons, 530 A3Es, and 232 A5Es (see Materials and Methods). Each of the A3Es and A5Es was represented by both “short” form (without the alternative extension) and “full” form (with the alternative extension).

**Figure 1:**
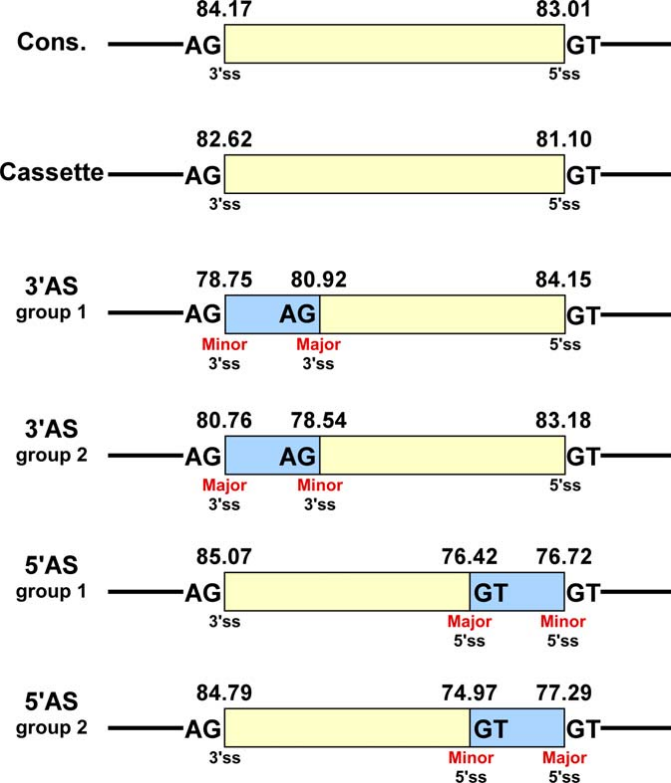
Special Characteristics of Alternative 3′ and 5′ Splicing Sites Human–mouse conserved 3′ and 5′ alternative splicing events (A3Es and A5Es, respectively) were divided into two subgroups according to their relative usage, in which the alternative splice site that is supported by most EST/cDNA is called Major, whereas the less-selected site is the Minor (see Materials and Methods). Splice site score of the 3′ and 5′ splice sites of constitutive, cassette (exon skipping), alternative 3′, and alternative 5′ exons was calculated using the “Analyzer Splice Tool” server (http://ast.bioinfo.tau.ac.il/SpliceSiteFrame.htm). Human exon scores are shown above the exon/intron junction scheme (see Figure S1 for mouse scores). Major/Minor splice site is indicated below each splice site. Exon sequence is represented by a yellow box and the alternative sequence (extension) by a light blue box. Introns are represented by black lines; canonical splice sites are shown in bold.

We first extracted the 3′ss and 5′ss of all the exons in the datasets and examined their strength (Figure 1). We found that while alternative cassette exons were shown to have relatively weaker splice sites in comparison with constitutive exons [32–35], A3Es and A5Es present a strong splice site in the fixated exon's side (i.e., 5′ in A3Es and 3′ in A5Es), resembling constitutive exons and statistically differing from alternative cassette exons (Mann-Whitney, *p* = 4.39E-05 and *p* = 8.67E-05 for A3Es and A5Es, respectively), and a weak splice site in the alternative exon's side (i.e., 3′ in A3Es and 5′ in A5Es). The alternative sites of A3Es and A5Es are even weaker than the splice sites flanking alternative cassette exons (Figure 1; see Tables 1 and 2 for statistical analysis and Figure S1 for mouse results). Thus, A3Es and A5Es contain a strong anchor at the constitutive splice site and suboptimal splice sites at the altered sites.

**Table 1:**
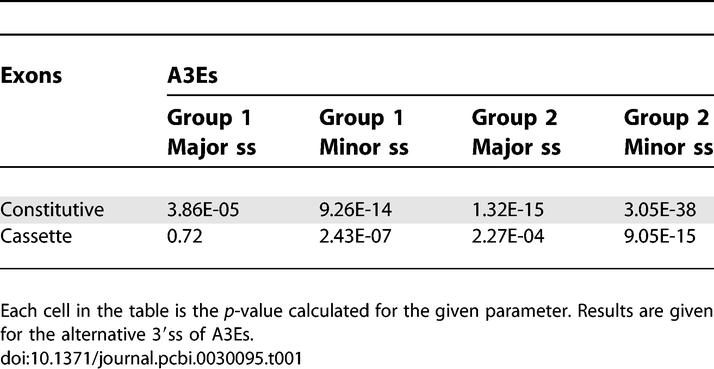
Statistical Analysis Results for A3Es' Splice Site Comparison Analysis

**Table 2:**
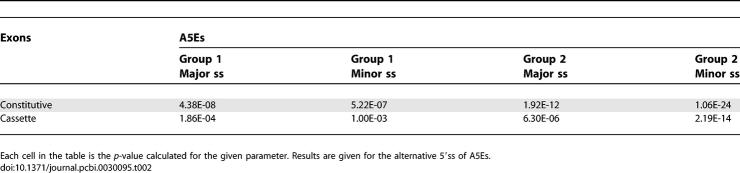
Statistical Analysis Results for A5Es' Splice Site Comparison Analysis

**Figure 2:**
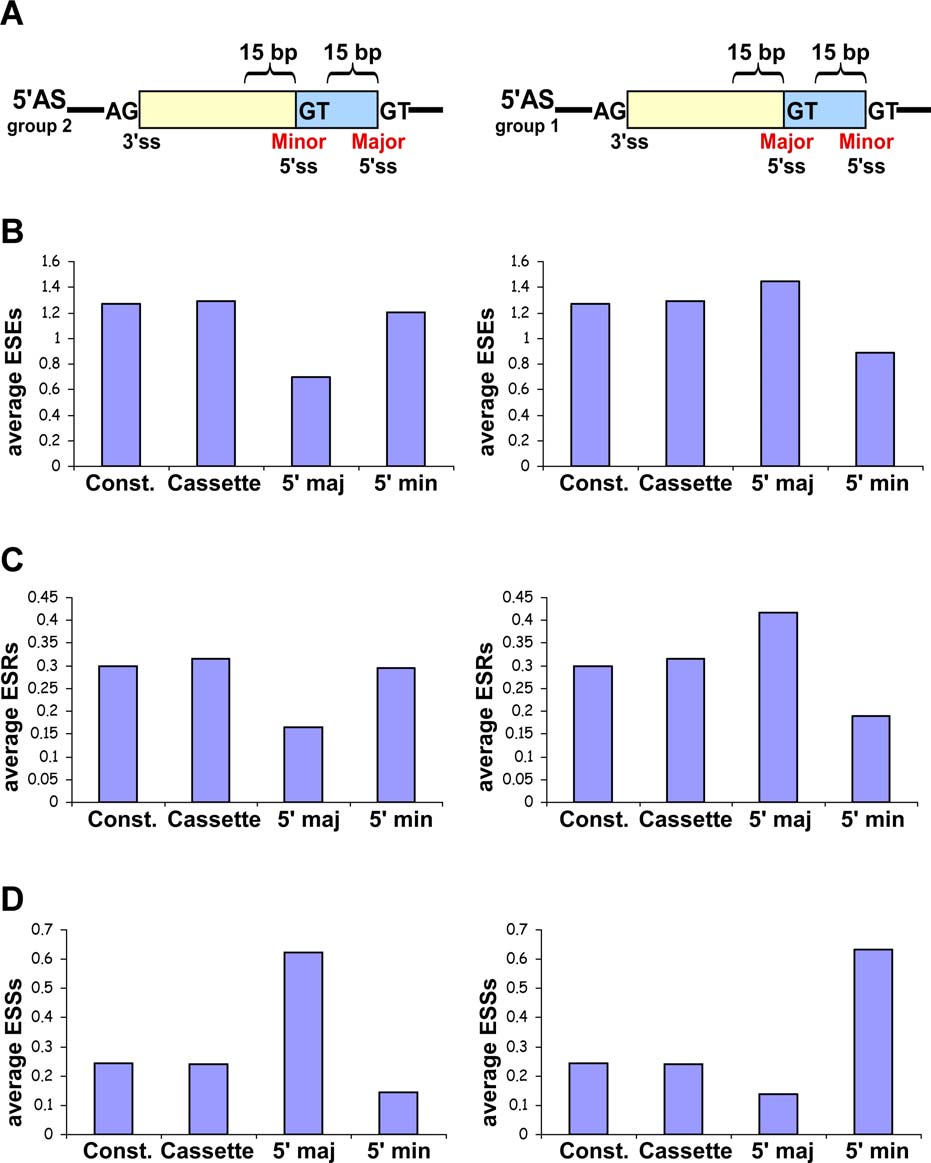
ESRs Analysis of Human–Mouse Conserved Alternative 5′ss Events A region 15 bp upstream of the major (5′ maj) and minor (5′ min) 5′ss was screened for ESRs. Average ESR number was calculated for each region. The analysis was performed for human–mouse conserved 5′ alternative splicing events that were divided into two subgroups according to their major/minor forms and the results were compared with constitutive (Const.) and alternative cassette conserved exons. Left and right panels are “group 2” (major form is longer than minor form) and “group 1” (major form is shorter than minor form) A5Es, respectively. (A) Schematic illustration of the analysis conducted. (B) Average ESEs [36]. (C) Average ESRs [10]. (D) Average ESSs [37].

Next, we tried to understand why the splicing machinery selects one splice site more frequently than the other, when the two are located on the same exon. We therefore determined which is the major and which is the minor splice site, based on ESTs coverage, requiring at least one transcript representing each of the forms (see Materials and Methods). The average number of cDNAs/ESTs per event is 78.27 and 56.12 cDNAs/ESTs for A3E and A5E, respectively. In addition, the percentage of events that were represented by a small number of cDNAs/ESTs is 1% and 2.3% for A3E and A5E, respectively. It is worth mentioning that we base this method of major/minor determination on the assumption that the cDNA/EST coverage of the splice forms properly represents the cellular situation. We subdivided each of the A3Es and A5Es into two subgroups, according to their extension inclusion level: (i) exons whose major splice site is within the exon—extension inclusion level is less than 40% (group 1); and (ii) exons whose major splice site is at the end of the exon—extension inclusion level is more than 60% (group 2). We observed that in A3Es (both group 1 and group 2) and in group 2 A5Es, the major splice site was significantly stronger than the minor splice site (Wilcoxon, *p* = 0.01, *p* = 2.37E-06, *p* = 0.007 for group 1 A3Es, group 2 A3Es, and group 2 A5Es, respectively). However, in the case of group 1 A5Es, both alternative splice sites had a similar splice site score. To examine this discrepancy further, we examined ESEs', ESSs', and ESRs' density in the 15 basepairs of the exonic sequences immediately upstream of the major and minor alternative 5′ss (Figure 2A), because it has been found that ESEs, ESSs, and ESRs in close proximity to the altered site have the major effect on alternative splicing [9–11]. It is noteworthy that even though the average distance between the two alternative splice sites is 63.71 (mean = 22), we cannot absolutely rule out the possibility that one regulatory element affects both alternative splice sites. Interestingly, we revealed that in the case of group 1 A5Es, there is a higher ESE density upstream of the major splice site than upstream of the minor splice site (Wilcoxon, *p* = 0.02 and *p* = 0.03 for ESRs and ESEs, respectively; Figure 2B and 2C, right panel). Conversely, in group 2 A5Es, an opposite trend can be observed, in which there is a higher ESE density upstream of the minor splice site than upstream of the major splice site (Wilcoxon, *p* = 0.001 and *p* = 0.02 for ESRs and ESEs, respectively). This phenomenon was demonstrated using both Fairbrother et al. ESEs and Goren et al. ESRs (Figure 2B and 2C, respectively, left panel, [10,36]). As opposed to ESEs, we found a lower ESS density upstream of the major splice site in group 1 exons and a very high density upstream of the minor splice site (Wilcoxon, *p* = 1.80E-04; Figure 2D, right panel; [37]). In group 2 exons, the results were vice versa, that is, there was a lower ESS density upstream of the minor splice site and a very high density upstream of the major splice site (Wilcoxon, *p* = 7.65E-08; Figure 2D, left panel). These results imply that A5Es require rigorous regulation for the proper selection of alternative splice sites, which, in the case of group 1 A5Es, assures the preferential selection of the major splice site despite the similar score of the two alternative sites. On the other hand, in the case of group 2 A5Es (in which the major splice site is stronger than the minor splice site), the regulatory mechanism is required to support minor splice site partial selection (see Figure S2 for mouse data). Thus, when the two alternative splice sites are of similar strength, a delicate interplay between ESE and ESS presumably directs splice site selection between the major and minor sites.

Further, we compared several additional features among the four datasets. Among the examined features were: exon length, conservation level between human and mouse (exon identity), divisibility-by-3 (symmetry), and K_A_/K_S_ ratio test. We found that the A3Es (both group 1 and group 2) show more resemblance to constitutive exons than to alternative cassette exons (Table 3). For instance, the average length of a constitutive exon is 132.46 bp (median = 121), which was found to be very similar to that of A3Es—136.6 (median = 128.5) and 147.02 (median = 129.5) for group 1 and group 2 exons, respectively. Statistically, the A3Es show no significant difference from constitutive exons. However, the difference from alternative cassette exons is significant (Mann-Whitney, *p* = 4.7E-07 and *p* = 1.04E-20 for group 1 and group 2 exons, respectively). Examination of the human–mouse conservation level showed the same trend. In this case, the A3Es are significantly different from constitutive exons (Mann-Whitney: *p* = 0.02 and *p* = 0.04 for group 1 and group 2 exons, respectively). However, the difference from alternative cassette exons is much more significant (Mann-Whitney, *p* = 2.16E-05 and *p* = 4.56E-20 for group 1 and group 2 exons, respectively). The symmetry analysis also showed significant difference from alternative cassette exons (χ^2^, *p* = 2.85E-09 and *p* = 4.69E-14 for group 1 and group 2 exons, respectively) and resemblance to constitutive exons (group 1 exons even presented lower levels of similarity).

**Table 3:**
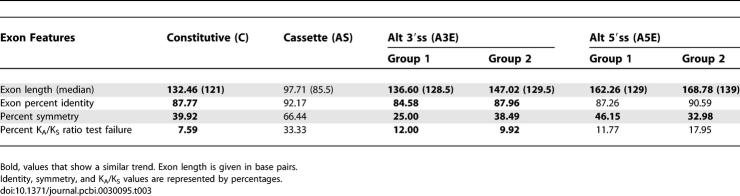
Features Comparison for Human–Mouse Conserved Exons (Whole Length Exons)

The K_A_/K_S_ ratio test was previously introduced as a tool for the identification of exons within genomic regions [38]. Briefly, the test examines whether the tested exon has evolved under purifying selection. A prospective research showed that this tool is more proficient in identifying constitutive exons rather than alternative cassette exons, which tend to fail the K_A_/K_S_ ratio test. Thus, it can serve as a discriminative tool between constitutive and alternative cassette exons [39]. As anticipated, constitutive and alternative cassette exons show significantly different K_A_/K_S_ ratio test failure percentages (7.59% and 33.33%, respectively; χ^2^, *p* = 8.51E-83). Regarding A3Es, 12% of group 1 exons and 9.92% of group 2 exons failed the test, showing resemblance to constitutive exons and a significant difference from alternative cassette exons (χ^2^, *p* = 0.41/0.33 and *p* = 0.03/4.67E-07 for group 1/group 2 versus constitutive and alternative cassette exons, respectively).

This same trend of resemblance to constitutive exons can also be observed in group 1 A5Es (group 1 exons) and in the length and symmetry features of group 2 A5Es (group 2 exons). However, in the case of group 2 A5Es, the identity level and K_A_/K_S_ ratio test features show more significant difference from the constitutive exons than from the alternative cassette exons (Table 3).

The analysis presented above shows that in the case of A3Es (both group 1 and group 2) and group 1 A5Es, all features examined indicate a resemblance to constitutive exons, whereas group 2 A5Es present inconsistent results (see Discussion).

We then checked the selected features in the extension sequence separately (the sequence between the two alternative splice sites). We found a high percentage of symmetry (71.19% and 63.81% for A3Es and A5Es, respectively), showing resemblance to alternative cassette exons and a significant difference from constitutive exons (χ^2^, *p* = 0.114 and *p* = 1.17E-42 for alternative cassette and constitutive exons, respectively). This is consistent with the notion that selective pressure is applied against the formation of an asymmetrical sequence between two alternative splice sites, because an asymmetrical sequence would shift the open reading frame and, hence, could lead to premature translation termination. Thus, the extension sequences show characteristics of cassette exons (Table 4).

**Table 4:**
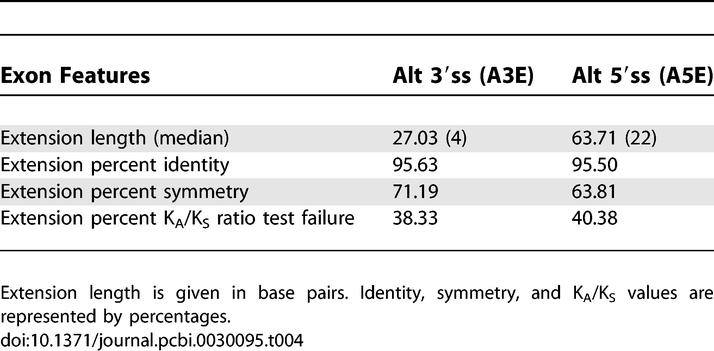
Features Comparison for Human–Mouse Conserved Exons (Extension Sequence)

### Conservation Level of Flanking Introns

**Figure 3:**
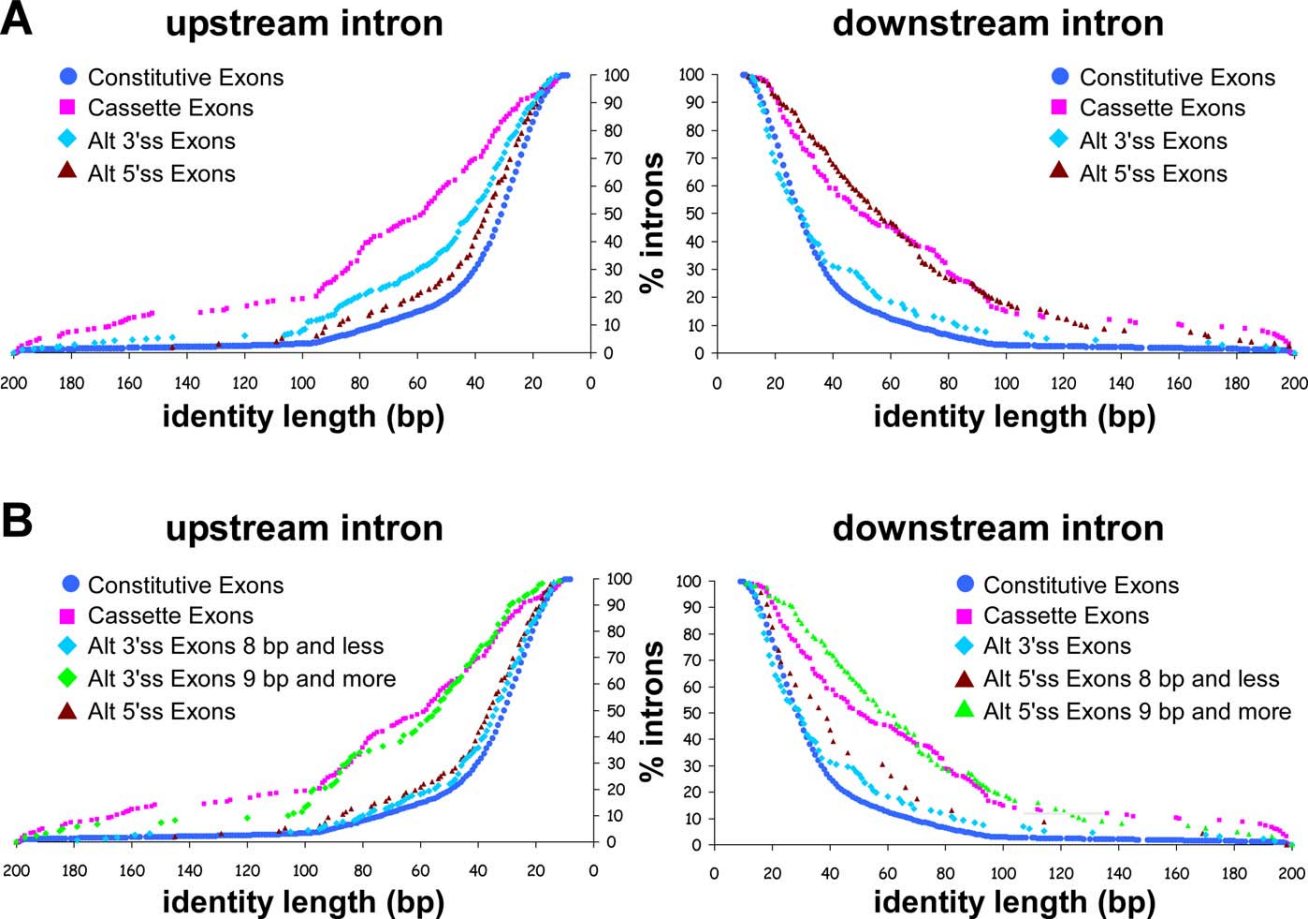
Flanking Introns Conservation Alignment of flanking intron regions was performed using the local alignment program Sim4 for upstream and downstream flanking intron (left and right panel, respectively). The *x*-axis represents the identity length (bp) and the *y*-axis represents the percentage of introns that were found to have an identity in that length or higher. (A) Conservation level of flanking introns for constitutive (blue circle), cassette (pink square), alt 3′ss (light blue diamond), and alt 5′ss (dark red triangle) exons. (B) Conservation analysis combined with division of the A3Es and A5Es into two subgroups according to their extension length (8 bp and less versus 9 bp and more). Analysis for the two subgroups is presented for the upstream intron of A3Es (light blue and light green diamonds) and for the downstream intron of A5Es (dark red and light green triangles).

Intronic sequences flanking constitutive exons show low conservation levels between human and mouse, whereas intronic sequences flanking alternatively spliced exons show 88% and 80% conservation of 103 and 94 bp on average for the upstream and downstream introns, respectively [40]. Also, tissue-specific cassette exons are flanked by highly conserved intronic sequences, even more conserved than cassette exons [41]. However, intronic sequences flanking the alternative side of A3Es and A5Es are characterized by a high conservation level, similar to alternative cassette exons, while intronic sequences flanking the fixated side are characterized by a relatively low conservation level, similar to constitutive exons [6]. A similar observation was also made on the NAGNAG motif subgroup of A3Es [20]. We thus examined the conservation of the intronic regions flanking A3Es and A5Es using a different method for the sequences alignment. Briefly, we used the local alignment program Sim4 to identify the human–mouse conserved regions. It is noteworthy that the intronic region flanking the alternative side was defined as the sequence downstream of the distal 5′ss (“most” downstream 5′ss) and upstream of the proximal 3′ss (“most” upstream 3′ss) for A5Es and A3Es, respectively (see Materials and Methods). Figure 3A demonstrates the percentage of upstream and downstream introns that are conserved between human and mouse (left and right panels, respectively). As expected, low conservation is observed in the intronic region flanking the fixated side (median length of 30 bp and 37 bp for A3Es and A5Es, respectively). However, there is a high conservation level in the intronic region flanking the alternative side (median length of 42 bp and 57 bp for A3Es and A5Es, respectively). Consequently, this analysis provides additional evidence for the importance of the intronic portion located immediately adjacent to the alternative site, whereas the fixated side is almost free from that constraint. Presumably, this conserved intronic sequence is involved in the subtle regulation of alternative splice site selection.

Another phenomenon that can be observed is that for A5Es, the conservation level in the alternative side is as high as in alternative cassette exons. However, A3Es show lower conservation levels in the alternative side, but higher than constitutive exons. To further examine this observation, we decided to divide the A3Es into two subgroups. It has been previously shown that an additional PPT appears only when the two alternative 3′ss are at least 8 bp apart [24]. Thus, we used this value as a cutoff for the subdivision. Surprisingly, repeating the analysis on the two subgroups showed that A3Es whose alternative splice sites are up to 8 bp apart show a conservation level similar to constitutive exons (average conservation length of 44.01 bp and average conservation level of 82.65), whereas A3Es in which the distance between the two 3′ss is longer than nine nucleotides show a conservation level that resembles alternative cassette exons (average conservation length of 69.39 bp and average conservation level of 86.16; Figure 3B, left panel; Figure S4A). We conducted the same analysis for A5Es using 6, 8, 9, and 20 bp as cutoffs, and the most significant change in the behavior of the two subgroups was observed using 8 or 9 bp as a cutoff (unpublished data). A5Es in which the distance between the two 5′ss is longer than nine nucleotides show a conservation level that resembles alternative cassette exons (average conservation length of 71.18 bp and average conservation level of 84.70), whereas the subgroup of A5Es whose alternative splice sites are up to 8 bp apart show a conservation level that is between the constitutive and alternative cassette exons (Figure 3B, right panel; Figure S4B). These results imply that there is a difference in the importance of the intronic regulatory elements according to the distance between the alternative splice sites: exons that present a long distance between the two competing splice sites rely on intronic sequences for proper alternative splice site selection, whereas exons that present a short distance between the two competing splice sites are relatively less dependent on those intronic sequences. These findings provide an additional interpretation to the regulatory implications of short distance alternative splice sites (see also [20,22,23]). Tables 5 and 6 summarize the entire statistical analysis. We also examined the flanking introns conservation level for group 1 and group 2 exons separately. However, no significant difference was found between these two subgroups (unpublished data), suggesting that selection of the major/minor splice site is not regulated via the intronic sequences.

**Table 5:**
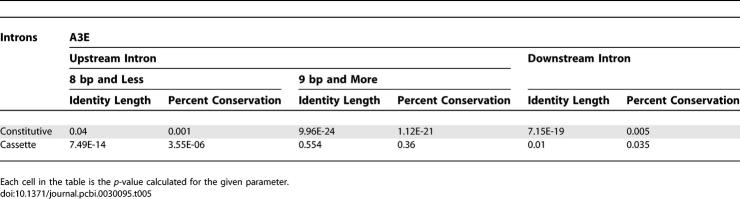
Statistical Analysis of A3Es' Flanking Introns Conservation

**Table 6:**
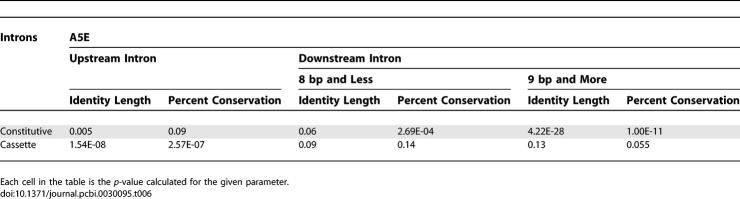
Statistical Analysis of A5Es' Flanking Introns Conservation

In addition, we observed a “local peak” in the conservation curve of the flanking introns around 75 bp and 60 bp for upstream and downstream introns, respectively, suggesting the preference of regulatory elements in these regions. Such a peak was previously reported in tissue-specific alternative cassette exons [41].

Based on the splice site score analysis, and the flanking intronic conservation, we suggest that A3Es and A5Es are a hybrid of constitutive and alternative cassette exons, where the alternative side of the exon (i.e., 3′ in A3Es and 5′ in A5Es) resembles alternative cassette exons, and the fixated side (i.e., 5′ in A3Es and 3′ in A5Es) resembles constitutive exons. The analysis of the characteristic features conducted on the exon as a whole showing resemblance to constitutive exons with the extension showing resemblance to alternative cassette exons further supports this hypothesis.

### *Alu* Elements Flanking Alternative 3′ and 5′ Exons

*Alu* elements are primate-specific retrotransposones, ~300 bp long, that have been shown to play a significant role in gene expression regulation [42]. Alternative cassette exons are flanked by longer intronic sequences, compared with constitutively spliced exons [5,43]. Density analysis of *Alu* sequences revealed that introns flanking alternative cassette exons demonstrate a significantly higher density of *Alu* sequences, compared with constitutive exons [44], which was also supported by our analysis (3.29/3.03 and 2.12/2 *Alu* sequences per intron for upstream/downstream introns of alternative cassette and constitutive exons, respectively. Mann-Whitney, *p* = 0.03/9.39E-05). When the density of *Alu* elements was examined in the flanking introns of A3Es, we found a resemblance to the constitutive exons with 2.44/1.98 *Alu* sequences for upstream and downstream introns, respectively (Mann-Whitney, *p* = 0.40 and *p* = 0.91 for upstream and downstream introns, respectively), which differs significantly from alternative cassette exons (Mann-Whitney, *p* = 0.048/0.02 for upstream and downstream introns, respectively). In contrast, A5Es showed an *Alu* density that resembles alternative cassette exons. However, these results were not significant.

### Evolution of A3Es and A5Es

**Figure 4:**
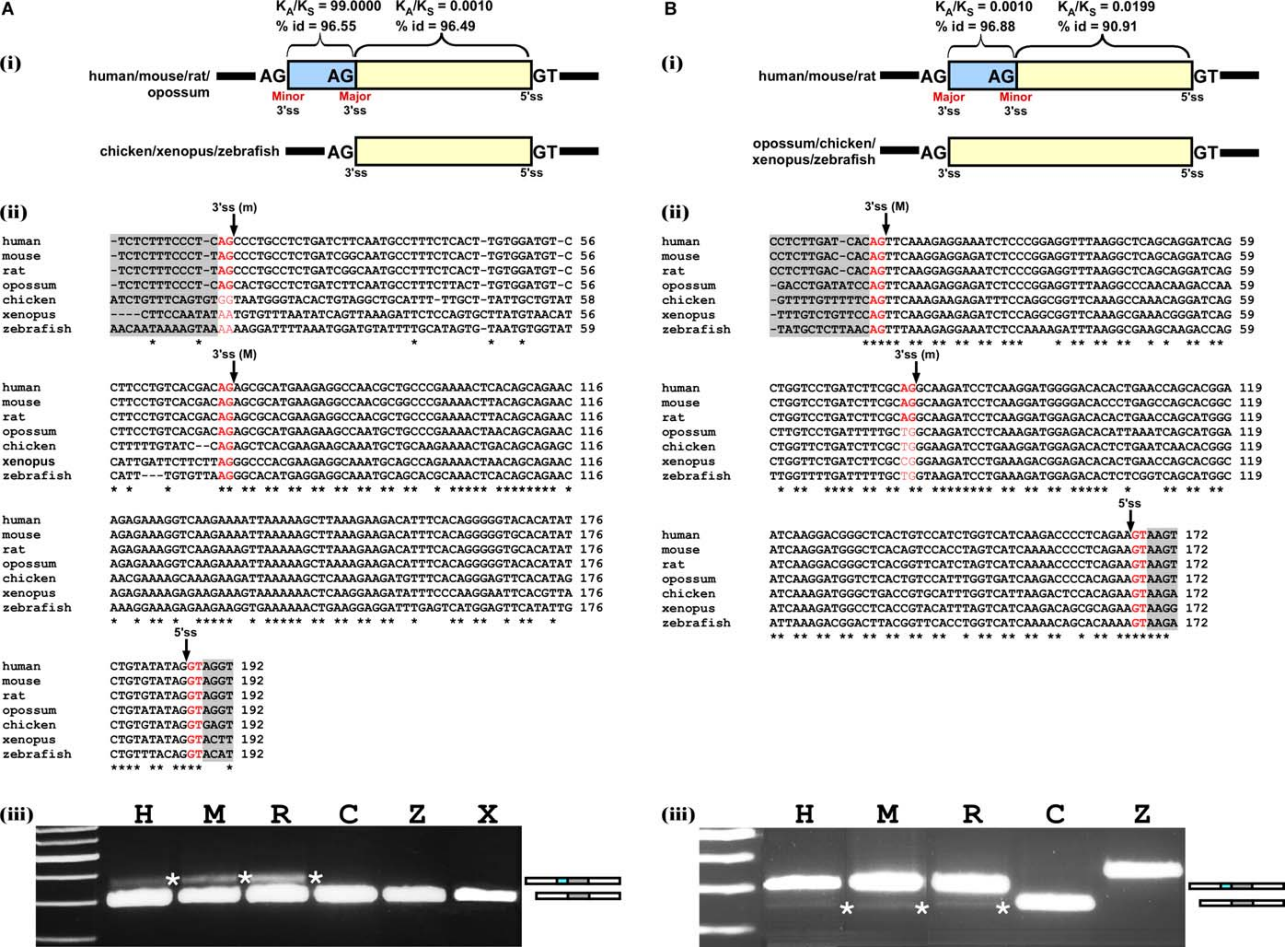
Mutations That Introduced a New Competing 3′ss Shifted Splicing from Constitutive to Alternative During Evolution Multiple alignment of homolog exons and flanking intronic sequences among seven vertebrate species was constructed according to a known evolutionary tree (see Table S1A for the sequences accessions) using ClustalW ([46]; http://www.ebi.ac.uk/clustalw). (A,B) The analysis was conducted on “group 1” and “group 2” alternative 3′ss exons, respectively. In the top panel (i) is a schematic representation of the handled exon. Exon sequence is represented by a yellow box and the alternative sequence (extension) by a light blue box. Introns are represented by black lines. The human–mouse K_A_/K_S_ values and identity percentage are shown above the boxes. Major and minor splice site is indicated below the boxes as well. The organisms that have a potential (according to their splice site content) to have either both alternatively spliced forms or only one of the forms are shown on the left of the schema. In the middle panel (ii) is a multiple alignment, among the seven species, of the exon and flanking 3′ and 5′ splice sites. The major 3′ss (M), minor 3′ss (m), and 5′ss are marked in red and pointed to by an arrow. Intronic regions are highlighted in light grey. On the bottom panel (iii) is an RT–PCR analysis on normal brain cDNA of human, mouse, and rat (H, M, and R, respectively), chicken 5-d embryo (C), adult zebrafish whole body (Z), and xenopus oocytes (X). PCR products were amplified using species-specific primers, and splicing products were separated on a 3% agarose gel and sequenced. Asterisks point to the alternative isoform; an illustration of both alternative isoforms is shown on the right by grey, cyan, and white boxes (alternative exon, extension, and flanking exons, respectively).

**Figure 5:**
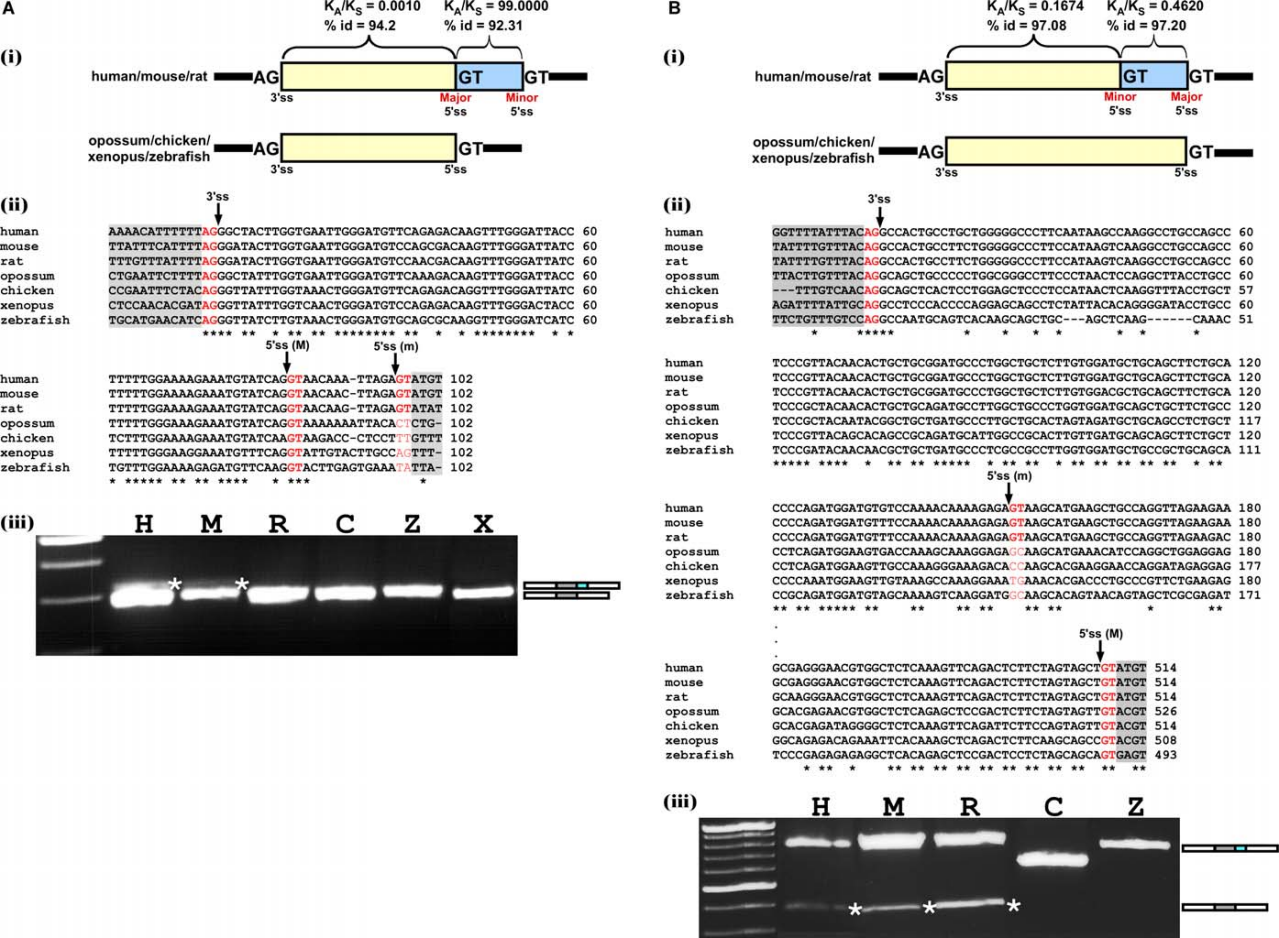
Mutations That Introduced a New Competing 5′ss Shifted Splicing from Constitutive to Alternative during Evolution Multiple alignment of homolog exons and flanking intronic sequences among seven vertebrate species was constructed according to a known evolutionary tree (see Table S1B for the sequences accessions) using ClustalW ([46]; http://www.ebi.ac.uk/clustalw). (A,B) The analysis was conducted on “group 1” and “group 2” alternative 5′ss exons, respectively. In the top panel (i) is a schematic representation of the handled exon. Exon sequence is represented by a yellow box and the alternative sequence (extension) by a light blue box. Introns are represented by black lines. The human–mouse K_A_/K_S_ values and identity percentage are shown above the boxes. Major and minor splice site is indicated below the boxes as well. The organisms that have a potential (according to their splice site content) to have either both alternatively spliced forms or only one of the forms are shown on the left of the schema. In the middle (ii) panel is a multiple alignment, among the seven species, of the exon and flanking 3′ and 5′ splice sites. The major 5′ss (M), minor 5′ss (m), and 3′ss are marked in red and pointed to by an arrow. Intronic regions are highlighted in light grey. On the bottom panel (iii) is an RT–PCR analysis on normal brain cDNA of human, mouse, and rat (H, M, and R, respectively), chicken 5-d embryo (C), adult zebrafish whole body (Z), and xenopus oocytes (X). PCR products were amplified using species-specific primers, and splicing products were separated on a 3.5% agarose gel and sequenced. Asterisks point to the alternative isoform; an illustration of both alternative isoforms is shown on the right by grey, cyan, and white boxes (alternative exon, extension, and flanking exons, respectively).

We conducted a human–mouse K_A_/K_S_ ratio analysis of the exonic region that is present in both the spliced variants (the short form, or core) of each exon, versus the extension sequence. The analysis was performed for group 1 and group 2 exons separately. Based on the K_A_/K_S_ ratio results, which indicated that the extension sequence behaves differently for group 1 and group 2 exons (Figures 4 and 5, panels (i)), we set to examine the evolutionary constraints affecting A3Es and A5Es. Altogether, we randomly selected and examined 13 cases for which we found the homologue exon and flanking exons sequences in the HomoloGene database among seven organisms (see below). Three of these cases showed putative functional alternative splice sites among all seven organisms, but no support was found for the alternative state for some of the organisms, probably because of a low EST/cDNA coverage of this region. In the remaining ten cases we found a functional alternative splice site only in some of the species. Figures 4 and 5 show four of such cases: one example representing a group 1 exon and one example representing a group 2 exon for both A3Es and A5Es. For each of the selected exons and flanking splice sites, we used the HomoloGene database to extract orthologous genes in seven different organisms [45]: human (Homo sapiens), mouse (Mus musculus), rat (*Rattus*), opossum (Didelphis virginiana), chicken (Gallus gallus), xenopus (Xenopus tropicalis), and zebrafish (Danio rerio); and generated a multiple sequence alignment among these seven species using ClustalW [46]. In group 1 A3E (human PRPF3 gene), we found the K_A_/K_S_ ratio of the major (short) form to be 0.001 (K_A_ = 0.0002, K_S_ = 0.1677) and the K_A_/K_S_ ratio of the extension to be 99 (K_A_ = 0.0476, K_S_ = 0.0005. Such a high ratio indicates that synonymous substitutions practically do not occur; Figure 4A, panel (i)). The multiple alignment of this exon revealed that the major (short) form is relatively conserved among the seven species (63.16%), while the extension showed poor conservation (13.33%). In addition, we found that at the minor splice site, a functional AG dinucleotide exists only among the mammals, whereas in chicken, xenopus, and zebrafish a nonfunctional 3′ss is present: either AA or GG dinucleotides in that site (Figure 4A, panel (ii)). We also observed that in human, mouse, rat, and chicken, a relatively strong PPT exists at the minor splice site, while in xenopus and zebrafish, no such sequence is present. Based on a known evolutionary tree, the most plausible scenario is that a mutation from A to G occurred, which created a functional AG 3′ss in human, mouse, rat, and opossum and resulted in the emergence of a new A3E. We noticed that no evidence (EST or cDNA) for alternative splicing was found for rat or opossum. However, this is likely a consequence of relatively small amounts of deposited mRNA and EST sequences that cover that locus.

Next, we validated these results experimentally in six of the seven species (human, mouse, rat, chicken, xenopus, and zebrafish). We designed primers for the flanking exons in each of these organisms. Total RNA was extracted from brain tissues of human, mouse, and rat, from a whole organism of chicken and zebrafish, and from xenopus' oocytes. This was followed by RT–PCR (reverse transcription–polymerase chain reaction) analysis and sequencing of all PCR products. The pairs of primers used in the RT–PCR were designed specifically to amplify a similar size of PCR product for the extension–inclusion isoform among the different organisms of the same ortholog. The experimental results validated that the alternative form was unique to human, mouse, and rat, which, as indicated above, have a functional minor splice site (Figure 4A, panel (iii); lanes H, M, R, which are abbreviations for human, mouse, and rat, respectively). In chicken, xenopus, and zebrafish, which were shown not to have an additional functional splice site, the exon is constitutively spliced (Figure 4A, panel (iii); panels C, Z, X, which are abbreviations for chicken, xenopus, and zebrafish, respectively). We note, that whenever the alternative 3′ss or 5′ss isoforms were not detected, longer exposure of the gels did not indicate its presence. However, this did not rule out completely that this alternative isoform (or selection of another cryptic splice site) might be detected under different conditions. Based on the phylogenetic relationships among the analyzed organisms and these results, we conclude that the alternative splice variant is a derived form, and the constitutive spliced variant is the ancestral one. Moreover, the constitutive form observed for chicken, xenopus, and zebrafish is also the major mRNA product of the human, mouse, and rat alternative forms. This indicates that the major form, which is the ancestral conserved isoform, is also the major mRNA product after the transition from constitutive to alternative splice site selection. Presumably, this is needed to prevent selection against the alternative isoform.

Unlike the group 1 A3E, in the group 2 A3E (human UBQLN4 gene), the K_A_/K_S_ ratio of the extension is similar to that of the minor (short) form (Figure 4B, panel (i)). The multiple alignment shows that the exon is conserved among the seven species throughout its full length (62.5%) and that only human, mouse, and rat present a functional minor splice site (AG), whereas opossum, chicken, and zebrafish contain TG in the reciprocal positions, and xenopus contains CG in the reciprocal positions. The minor splice site is within the exon, which is relatively conserved among the seven species. Thus, we find a relatively strong PPT at the minor splice site in all seven species. The minor 3′ss was presumably created during evolution following a mutation in the first position of the AG 3′ss (Figure 4B, panel (ii)). The experimental results verify that only the human, mouse, and rat present two alternatively spliced forms, which is compatible with the bioinformatic analysis, while the chicken and zebrafish (no PCR product was observed for xenopus) present only one constitutive splice form (Figure 4B, panel (iii)). The different size of the chicken PCR product is due to the fact that the forward primer was designed on the A3E, because the upstream exon does not exist in the University of California Santa Cruz Genome (UCSC) Browser (see Table S1). Again, the major mRNA product is the major form, which is the constitutive and only mRNA product in chicken and zebrafish, suggesting that it is the ancestral form. Based on the phylogenetic relationships among the analyzed organisms, we concluded that the alternative splice variant is a derived form, and the constitutive spliced variant is the ancestral one. A corresponding trend can be observed in group 1 A5E (human ACTR6 gene; Figure 5A) and group 2 A5E (human NCOR1 gene; Figure 5B). The chicken PCR product is different in size, because the forward primer was designed on the A5E, as the upstream sequence is not fully mapped in the UCSC Genome Browser (see Table S1).

The tested exons are alternatively spliced in human, mouse, and rat. In these species, the ancestral form is also the major mRNA product, but these exons are constitutively spliced in chicken, xenopus, and zebrafish (Figures 4 and 5, panel (iii)). Based on the phylogenetic relationships among the analyzed organisms and the above results, we concluded that the examined cases of A3Es and A5Es originated from a constitutive ancestral form (originally including the alternative extension or excluding it). Mutations upstream or downstream of the ancestral splice site created a new functional splice site that competes with the original splice site, but with a lower selection level. This competition results in an alternative selection between those sites. This hypothesis is consistent with the high level of similarity found between A3Es and A5Es and constitutive exons. It is worth mentioning that although we only demonstrated four cases, in which the origin of alternative 3′ss and 5′ss is from previously constitutively spliced exons, we believe that since these cases are representatives of the four exon groups, and their characteristics apply to all exons, we thus suspect that a major portion of the A3Es and A5Es originated by this process.

## Discussion

Here we used bioinformatic tools to examine A3Es and A5Es that are conserved between human and mouse and, thus, are potentially functionally meaningful events. We divided each of the A3Es and A5Es datasets into two subgroups: one in which the major splice form is spliced using the splice site within the exon (group 1) and the other in which the major splice form is spliced using the splice site at the end of the exon (group 2), according to each of their alternative forms inclusion level. We used these four subgroups to examine unique characteristics that distinguish these types of splicing events. It has been previously shown that constitutive and alternative cassette exons differ in several features, such as exon length, conservation level between human and mouse (exon identity), divisibility-by-3 (symmetry), and K_A_/K_S_ ratio test [6,14,29,39]. We demonstrated that A3Es and A5Es, as a whole, show a resemblance in the aforementioned characterizing features to constitutive exons and differ from alternative cassette exons. These findings are statistically significant for group 1 and group 2 A3Es, as well as for group 1 A5E exons.

Further, we showed that when examining splice site strength, flanking introns conservation, and *Alu* density in the fixated side (i.e., 5′ in A3Es and 3′ in A5Es) and the alternative side (i.e., 3′ in A3Es and 5′ in A5Es) separately, we find that the fixated side shows a resemblance to constitutive exons, whereas the alternative side shows a resemblance to alternative cassette exons. Thus, we suggest that A3Es and A5Es can be regarded as an intermediate state between constitutive and alternative cassette exons. Moreover, we showed that the alternative side of the A5Es requires precise regulation, presumably to achieve proper splice site selection. Previously, it was shown that ESE and ESS elements have the major effect on splicing when located in proximity to the alternative splice site [9–11]. In the case of group 1 A5Es, this regulation is essential, because both alternative splice sites are of similar strength; high ESE/ESR and low ESS density in proximity to the major splice site are probably the major factors that govern the level of each site usage in splicing. On the other hand, in group 2 A5Es, the major splice site is stronger than the minor splice site. Thus, the ESE/ESR density is higher, and the ESS density is lower in proximity to the minor splice site. This provides relative predominance and enables the selection level of the minor splice site and, thus, presumably regulates alternative splice site selection. It is noteworthy that examination of the ESR density in proximity to the alternative splice sites in A3Es found no significant results (unpublished data). This may be because ~50% of A3Es belong to the NAGNAG family [20,21,24], and, thus, the alternative forms differ from each other by only 3 bp, which makes the region scanned for ESRs overlap between the two alternative forms. Also, it is possible that, in A3Es, the ESRs play a minor role in the decision of major/minor 3′ss selection and that screening downstream of the branch site determines alternative 3′ss usage [47,48].

Relying on the assumption that sequence conservation implies functional properties, we conducted an analysis on the conservation of flanking intronic sequences. In agreement with other reports [6,20], we showed that the conservation level of the intronic sequence flanking the alternative side resembles the conservation level observed in alternative cassette exons. This analysis also revealed that, while the conservation level in the downstream intron flanking A5Es is relatively similar to alternative cassette exons, the conservation level of the upstream intron flanking A3Es is in between the conservation level of constitutive and alternative cassette exons. To resolve this discrepancy, we decided to divide the A3Es dataset into two subgroups that are secluded by the length of the extension sequence. The chosen cutoff was 8 bp, which is based on a previous report showing that an additional PPT appears only when the two alternative splice sites are at least 8 bp apart [24]. We were intrigued to find that the subgroup whose extension is 8 bp or less presents a low intronic conservation level that resembles constitutive exons, while the subgroup whose extension is 9 bp or larger presents a high intronic conservation level which resembles alternative cassette exons. This suggests that regulation via the intronic sequences on alternative 3′ss occurs only when the alternative splice sites are not in close proximity, whereas selection of splice sites that are located in close proximity may be “noise” of the splicing mechanism [23]. We conducted the same analysis on A5Es and found that the subgroup of exons whose extension is 8 bp or less shows a conservation level that is between constitutive and alternative cassette exons, and the other subgroup showed a high conservation level that is similar to (and even higher than) alternative cassette exons (Figure 3 and Tables 5 and 6).

The K_A_/K_S_ ratio is used for the estimation of the selective forces acting on proteins [14]. That is, K_A_/K_S_ ≪ 1 indicates purifying selection and K_A_/K_S_ ≫ 1 indicates positive selection. We used this estimation tool to examine whether the selective forces throughout evolution acted in a different way on the core exon (i.e., the exon sequence not including the region that is alternatively spliced) compared with the alternative region (i.e., the region which is either included or not). We performed the analysis for the subgroup of exons for which the major splice site is within the exon (group 1) and for the subgroup of exons for which the major splice site is at the end of the exon (group 2) separately. In both group 1 and group 2 exons, the core exon presented K_A_/K_S_ ≪ 1, indicating high purifying selection. In group 1 exons, the extension (i.e., the alternative region) generally presented K_A_/K_S_ ≫ 1, indicating that it is under positive selection, so that the extension form is free to evolve and eventually potentially acquire a new function. In contrast, in the case of group 2 exons, the alternative region generally presented K_A_/K_S_ ≪ 1, which was relatively similar to the core K_A_/K_S_, indicating that the same selective forces have acted on both regions. This raises the possibility that, in the case of group 1 exons, the extension region was newly introduced during evolution, while in the case of group 2 exons the extension region was part of the original exon.

Based on this analysis, we suggest that dynamic evolution is the key for the emergence of a new A3E or A5E. We propose that a certain fraction of the A3Es and A5Es originated from a previously constitutively spliced exon that acquired a competing splice site upstream or downstream of the ancestral authentic splice site. As a result, both sites are selected, thus giving birth to an alternative 5′ss or 3′ss exon. We believe that selective forces acted to ensure the symmetry of the created alternative region (the region between the authentic splice site and newly created splice site), to maintain the same reading frame as that of the ancestral form, presumably by the selection of events that ensure this symmetry. We also assume that, initially, the selection of the new splice site was very rare and lacked biological functionality, but during the course of evolution such functionality was acquired, in a process that is called exaptation [49]. Thus, an ancestral form including the extension (i.e., alternative splice site is within the original ancestral exon sequence) will show a similar K_A_/K_S_ ratio throughout the exon, because the same evolutionary purifying selection forces took effect before and after the appearance of the alternative splice site. However, an ancestral form excluding the extension (alternative splice site is inside the flanking intron) will show a higher K_A_/K_S_ ratio in the extension (alternative added sequence) than in the original short form, because the evolutionary purifying selection forces began operating at a much later time. We have shown that in four tested features, the A3Es and A5Es are similar to constitutive exons (see above). However, in one subset of group 2 A5Es, the exons are more similar in their conservation level and in their K_A_/K_S_ ratio to alternative cassette exons than to constitutive exons. We believe that the evolutionary model described previously can also serve as a possible explanation for these results. That is, the alternative 5′ss appeared during evolution within exons that were already under purifying selection to maintain the coding sequence. Thus, the exon that includes the alternative region is the evolutionary ancient one, and the exon that excludes the alternative region is the new addition. Another possibility is that A3Es and A5Es can also originate from alternative cassette exons in which one of the splice sites (5′ss in A3Es and 3′ss in A5Es) is strengthened, because of mutations, and an existing or a newly generated cryptic splice site began to be selected as an alternative splice site.

We decided to examine our hypothesis of constitutive origin on a limited number of exons manually; one example represented a group 1 exon and one example represented a group 2 exon for both A3Es and A5Es. We used multiple species alignment of seven species for each of the selected exons and their flanking splice sites to pinpoint the molecular evolutionary changes. The multiple alignments show that while in chicken, xenopus, and zebrafish only one functional splice site can be observed (AG and GT for 3′ss and 5′ss, respectively), in human, mouse, rat, and, sometimes, in opossum, another alternative functional splice site was created by a mutation during evolution. It suggests that, based on a known evolutionary tree (see Figure S3), the appearance of new functional alternative splice sites as a result of mutations in the discussed examples is mammal-specific (not necessarily in all mammals). We further validated these cases experimentally, showing that the most plausible scenario is that A3Es and A5Es were originated from previously constitutive exons. In these exons, an alternative splice site emerged in the lineage leading to mammals, following a mutation that created a functional 3′ss or 5′ss (AG or GT, respectively). After the creation of the alternative site, selection pressures show differences between sites created outside of the ancestral exon or within the ancestral exon. In the case of group 1 exons relative to the ancestral form, the extension is poorly conserved (between the seven species) and presents K_A_/K_S_ ≫ 1, while in the group 2 exons, the extension is relatively highly conserved and presents K_A_/K_S_ < 1. Although our analysis experimentally validates the hypothesis for these cases, a broader evolutionary examination and large-scale analysis is still required to examine the scope and magnitude of this phenomenon.

In conclusion, we examined the characteristics of A3Es' and A5Es' splicing forms, showing that A3Es and A5Es contain an anchor splice site that is as strong as that of constitutive exons and alternative sites that are weaker than cassette exons. We propose an evolutionary dynamic model in which A3Es and A5Es originated from ancestral constitutive exons that following mutation/s, a new alternative splice site appeared, and started competing with the ancient one for splice site selection. This model is supported by bioinformatic analysis showing that A3Es and A5Es are similar to constitutive exons. It was validated for four exons by multiple species' comparison and experimental validation. We also present that when two alternative 5′ss are of a similar strength, a delicate ratio between ESE/ESS located immediately upstream of each splice site determines which one is the major or minor selected site. In addition, A3Es and A5Es whose alternative splice sites are at least 9 bp apart show a high intronic sequences conservation level, indicative of the participation in the splicing regulation of these exons.

## Materials and Methods

### Dataset compilation.

We assembled a unified dataset of human–mouse orthologous exons [6,30,31]. The dataset included 45,553 constitutively spliced exons, 757 cassette exons, 530 A3Es, and 232 A5Es. For each of the A3Es and A5Es, obtained from Sugnet et al. (2004), we extracted the DNA and mRNA sequences using the BLAST program and produced an exon/intron structure using the Sim4 program. The Sim4 program receives a DNA sequence and an mRNA sequence and projects the mRNA onto the DNA considering the exon/intron architecture and the flanking consensus splice sites. This led to acquisition of both forms of A3Es and A5Es, including their respective flanking intronic sequences. For the Carmel et al. (2004) and Sorek et al. (2004) datasets, this information was already extracted.

### Establishing major/minor form subgroup division.

The dataset of A3Es and A5Es was divided into two subgroups: (i) exons whose major splice site is within the exon and (ii) exons whose major splice site is at the end of the exon. These subgroups were defined as “group 1” and “group 2,” respectively (Figure 1). The determination of whether an exon is in one subgroup or another was made by locating all the mRNA and EST sequences hits received from blasting that exon and the flanking exons against all the sequences in the UCSC database (http://genome.ucsc.edu), requiring at least one transcript representing each form. The number of long forms, i.e., those that include the alternative region (N_L_), versus short forms, i.e., those that exclude the alternative region (N_S_), were counted, and the extension inclusion percentage (i.e., the fraction of mRNAs and ESTs containing the alternative region) was calculated as [N_L_ / (N_L_ + N_S_)] * 100. Exons whose extension inclusion percentage was less than 40% were attributed to “group 1,” and exons whose extension inclusion percentage was more than 60% were attributed to “group 2.” Exons whose extension inclusion percentage was in the range of 40% to 60% were not included in either group, so as to prevent false predictions resulting from a low EST coverage or borderline cases.

### Splice site score analysis.

3′ss and 5′ss scores were extracted using a program based on the “Analyzer Splice Tool” server (http://ast.bioinfo.tau.ac.il/SpliceSiteFrame.htm). The program was adjusted for multisequence analysis, using the algorithm of Shapiro and Senapathy (1987). Only canonical splice sites (i.e., AG in positions −2 and −1 for 3′ss and GT in positions +1 and +2 or GCA in positions +1 to +3 for 5′ss) were considered. The analysis was executed for the human and mouse splice sites separately.

### Calculation of flanking introns identity percentile.

A region of 100 bp for each flanking intron was selected. The intronic region flanking the alternative side was defined as the sequence downstream of the distal 5′ss (“most” downstream 5′ss) and upstream of the proximal 3′ss (“most” upstream 3′ss) for A5Es and A3Es, respectively. A homology examination was performed using Sim4 [50] with its default parameters. Briefly, this program detects exact matches of length 12 and extends them in both directions with a score of 1 for a match and −5 for a mismatch, stopping when extensions no longer increase the score. For homology of 100 bp long, the following 100 bp were also examined (and so on). The length of the homology was defined as the sum of lengths of the homology regions identified by Sim4 (L_1..n_), and the weighted identity percentage was calculated as (Σ_i=1..n_ L_i_ * I_i_) / Σ_i=1..n_ L_i_, where I_i_ is the identity percentage for the I'th homology region. The relative percentile was calculated for each of the sequences. The A3Es and A5Es datasets were then both divided into two subgroups, according to the length of the extension (number of bp between the alternative splice sites) when the cutoff used was 8 bp. The analysis was then also conducted for each subgroup separately.

### Multispecies alignment of alternatively spliced exon and flanking splice sites.

The exon and flanking splice site sequences of rat, opossum, chicken, xenopus, and zebrafish were extracted, using the UCSC Genome Browser (http://genome.ucsc.edu). The sequences of the seven species (including human and mouse) were aligned using the ClustalW program, with its default parameters ([46], http://www.ebi.ac.uk/clustalw). Major and minor (authentic—i.e., supported by mRNAs or ESTs; potential—i.e., not supported by mRNAs and ESTs and noncanonical) 3′ss and 5′ss were located and marked.

### Total RNA isolation and RT–PCR amplification.

Chicken (5-d embryo) and zebrafish (adult) were disrupted in TRIzol (Sigma, http://www.sigmaaldrich.com) with a Polytron homogenizer (PT-MR2100 Kinematica, http://www.kinematica.ch), or with a hand-held motor-pestle (Kimble-Kontes, http://www.kimble-kontes.com) for the xenopus' oocytes. After complete homogenization of the tissue, the total RNA was isolated. The samples were treated with 2U of RNase-free DNase (Ambion, http://www.ambion.com). RT was performed on 1–2 μg total RNA using RT–AMV (avian myeloblastosis virus, Roche, http://www.roche.com) following the manufacturer's protocol. We used commercial brain cDNA for human, mouse, and rat (BioChain, http://www.biochain.com). Endogenous PCR amplification was performed, using Taq polymerase (BioTools, http://www.btools.com) and species-specific primers (see Table S2 for the sequences of the primers). Amplification was performed for 30 cycles, consisting of denaturation for 30 s at 94 °C, annealing for 45 s at the appropriate Tm, and elongation for 1–2 min at 72 °C. The spliced cDNA products were separated in 3%–3.5% agarose gel. We note that, in the case of UBQLN4 (Figure 4B (iii)) and NCOR1 (Figure 5B (iii)) exons, the RT–PCR failed to amplify the cDNA of the xenopus. All PCR products were eluted and sequenced.

## Supporting Information

Figure S1 Special Characteristics of Alternative 3′ and 5′ Splicing Sites Human–mouse conserved 3′ and 5′ alternative splicing events (A3Es and A5Es, respectively) were divided into two subgroups according to their major/minor selected sites (see Materials and Methods). Splice site score analysis of the 3′ and 5′ splice sites of constitutive, cassette (exon skipping), alternative 3′, and alternative 5′ exons was conducted. Human and mouse exon scores are shown above the exon/intron junction scheme (mouse scores are below the human scores). Major/Minor splice site is indicated below each splice site. Exon sequence is represented by a yellow box and the alternative sequence (extension) by a light blue box. Introns are represented by black lines, canonical splice sites are shown in bold.
(264 KB PDF)

Figure S2 ESRs Analysis of Human–Mouse Conserved Alternative 5′ss Events A region 15 bp upstream of the major (5′ maj) and minor (5′ min) 5′ss was screened for ESRs. Average ESR number was calculated for each region. The analysis was done for human–mouse conserved 5′ alternative splicing events that were divided into two subgroups according to their major/minor forms (see Materials and Methods) and compared to constitutive (Const.) and alternative cassette conserved exons. Left and right panels are “group 2” (major form is longer than minor form) and “group 1” (major form is shorter than minor form) A5Es, respectively. Human and mouse results are in light blue and purple, respectively.
(A) Schematic illustration of the analysis conducted.
(B) Average ESEs [36].
(C) Average ESRs [10].
(D) Average ESSs [37].
(323 KB PDF)

Figure S3 Evolutionary Tree of the Seven Analyzed Species A phylogenetic evolutionary tree spanning the seven analyzed species built based on previous evolutionary studies [51].
(102 KB PDF)

Figure S4 Flanking Introns Average Conservation Length Alignment of flanking intron regions was performed using the local alignment program Sim4 for upstream and downstream flanking intron (A and B, respectively). The *x*-axis represents the group of exons and the *y*-axis represents the average conservation length (bp).
(180 KB PDF)

Table S1 Accession Numbers of the Seven Analyzed Vertebrate Species (39 KB DOC)

Table S2 Primers Used in the Experimental Validation (58 KB DOC)

### Accession Numbers

Accession numbers from the National Center for Biotechnology Information (http://www.ncbi.nlm.nih.gov/RefSeq) are: Homo sapiens PRPF3 (NM_004698), Gallus gallus (NM_001031390), Homo sapiens UBQLN4 (NM_020131), Mus musculus (NM_033526), Gallus gallus (NM_001031373), Xenopus tropicalis (NM_001037720), Danio rerio (NM_213356), Homo sapiens ACTR6 (NM_022496), Xenopus tropicalis (NM_001016472), and Homo sapiens NCOR1 (NM_006311).

Accession numbers from The National Center for Biotechnology Information GenBank (http://www.ncbi.nlm.nih.gov/Genbank) are Mus musculus (BC026607), *Rattus* (CB586372), Xenopus tropicalis (CX437471), Danio rerio (CK028262), *Rattus* (CB747083), Mus musculus (CK617284), *Rattus* (CK365697), Danio rerio (BC045961), *Rattus* (CB546074), Gallus gallus (BU326580), Xenopus tropicalis (BC099620), and Danio rerio (CK015548).

The accession number from the EMBL Nucleotide Sequence Database (http://www.ebi.ac.uk/embl) is Gallus gallus (AJ719762).

The accession number from the DNA Data Bank of Japan (http://www.ddbj.nig.ac.jp) is Mus musculus (AB093281).

## Abbreviations

A3E: alternative 3′ splice site exon
A5E: alternative 5′ splice site exon
ESE: exonic splicing enhancer
ESR: exonic splicing regulatory element
ESS: exonic splicing silencer
EST: expressed sequence tag
PCR: polymerase chain reaction
PPT: polypyrimidine tract
RT: reverse transcription
ss: splice site
