# Supplementary figures and images for: The Emergence of Alternative 3′ and 5′ Splice Site Exons from Constitutive Exons
Source: PLoS Comput Biol. 2007 May 25;3(5):e95. doi: 10.1371/journal.pcbi.0030095 (PMC1876488; doi:10.1371/journal.pcbi.0030095)

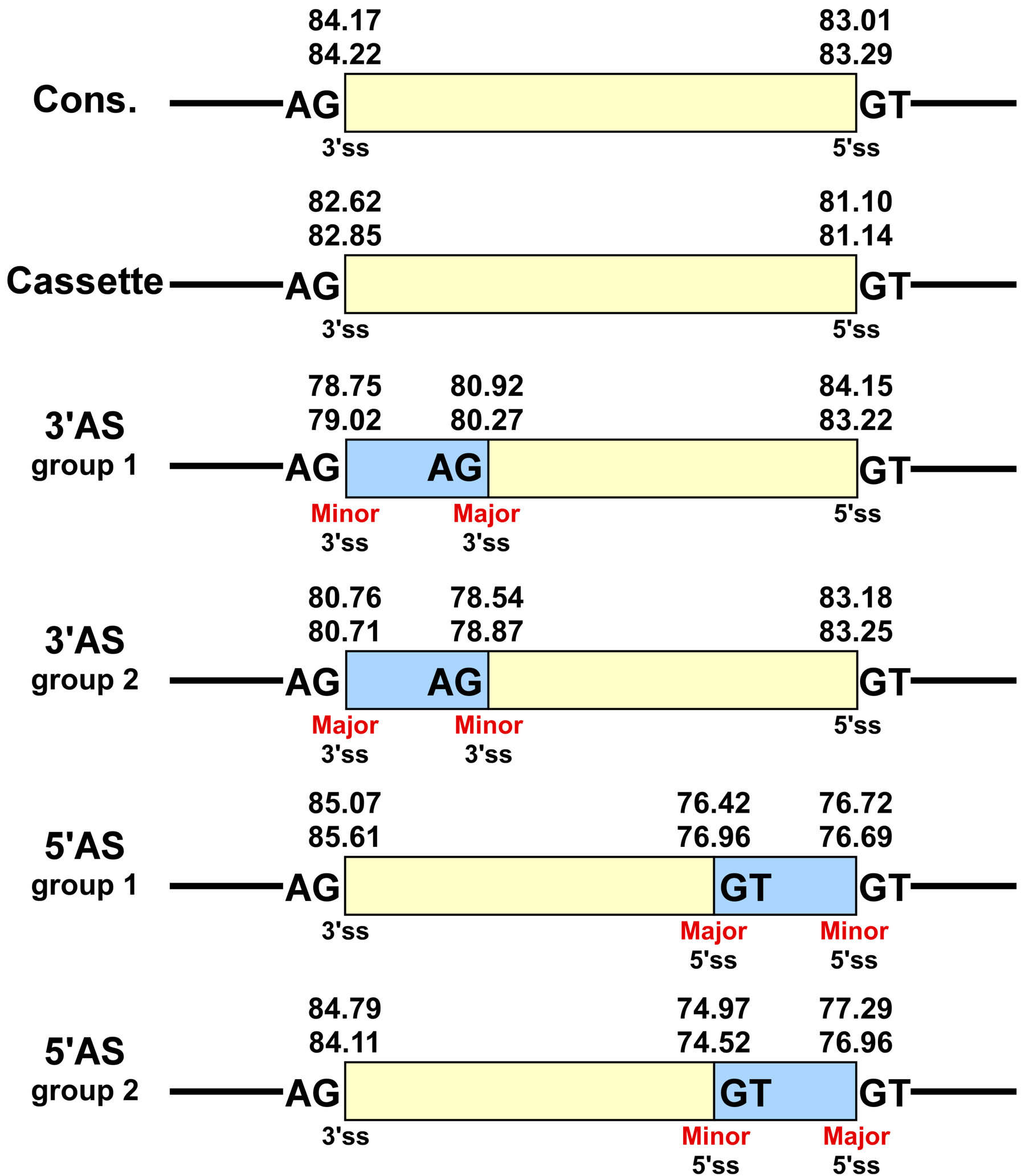

Figure S1

Supplement: Figure S1 — Human–mouse conserved 3′ and 5′ alternative splicing events (A3Es and A5Es, respectively) were divided into two subgroups according to their major/minor selected sites (see Materials and Methods). Splice site score analysis of the 3′ and 5′ splice sites of constitutive, cassette (exon skipping), alternative 3′, and alternative 5′ exons was conducted. Human and mouse exon scores are shown above the exon/intron junction scheme (mouse scores are below the human scores). Major/Minor splice site is indicated below each splice site. Exon sequence is represented by a yellow box and the alternative sequence (extension) by a light blue box. Introns are represented by black lines, canonical splice sites are shown in bold. (264 KB PDF) [file pcbi.0030095.sg001.pdf]

**A**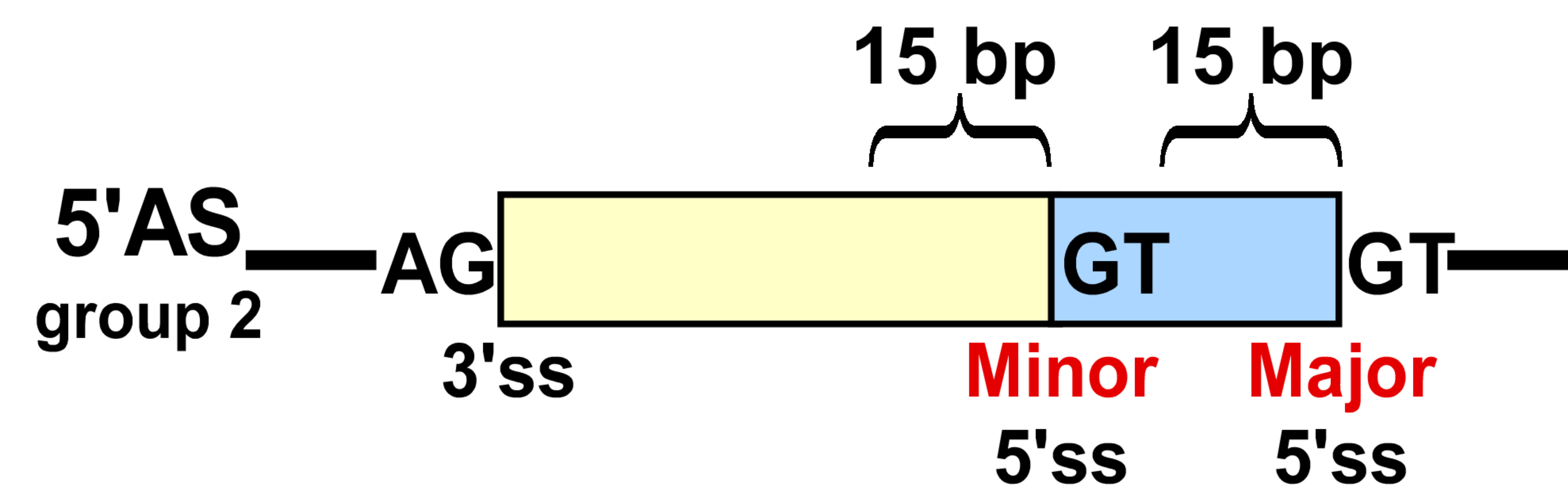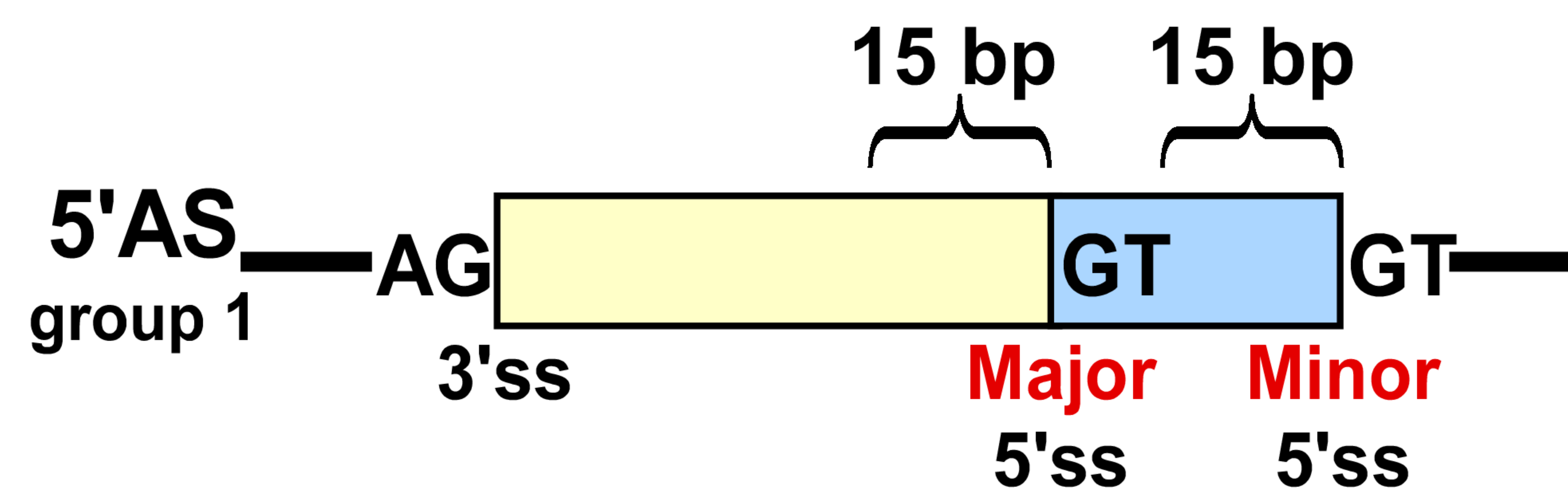**B**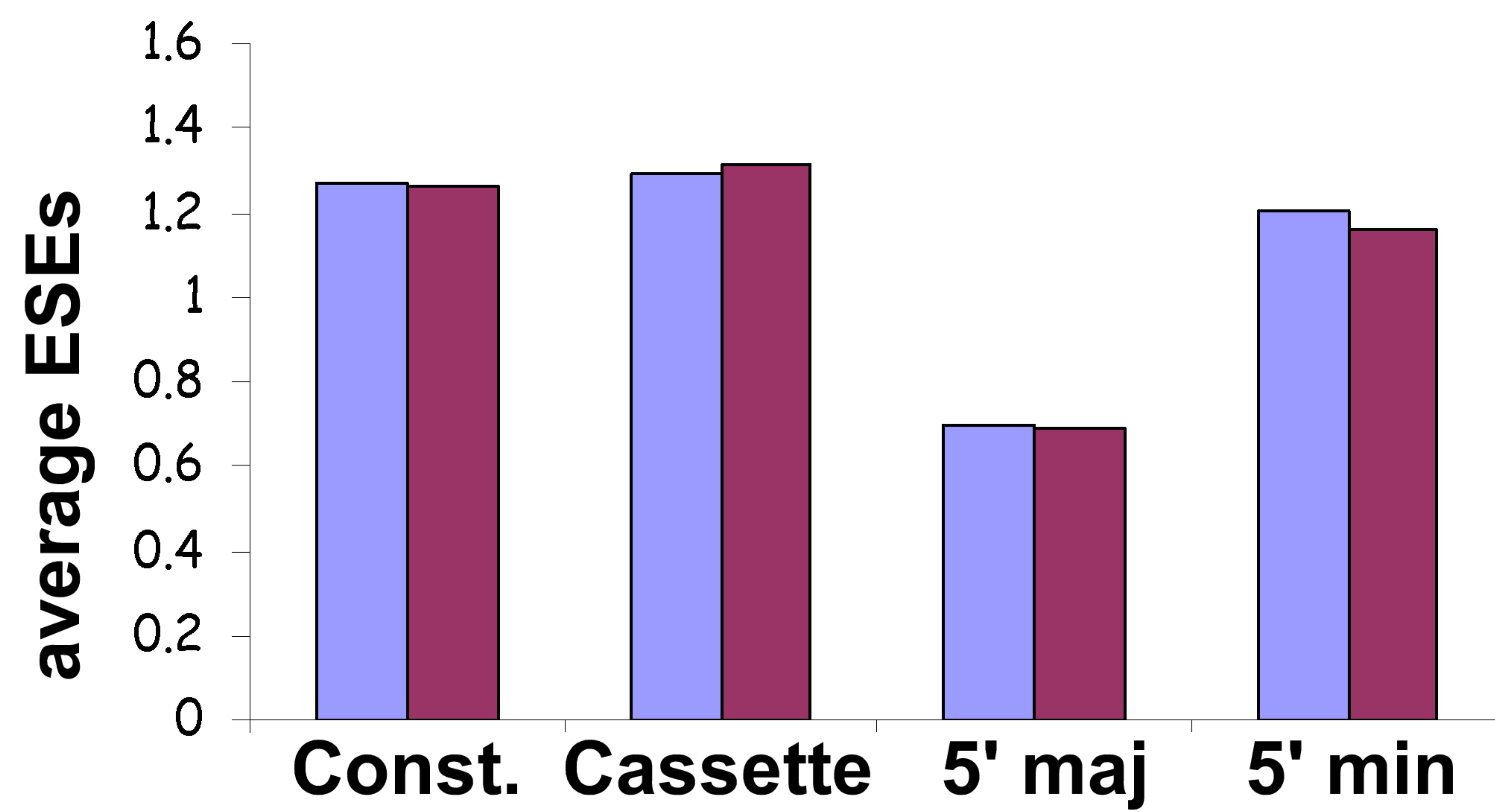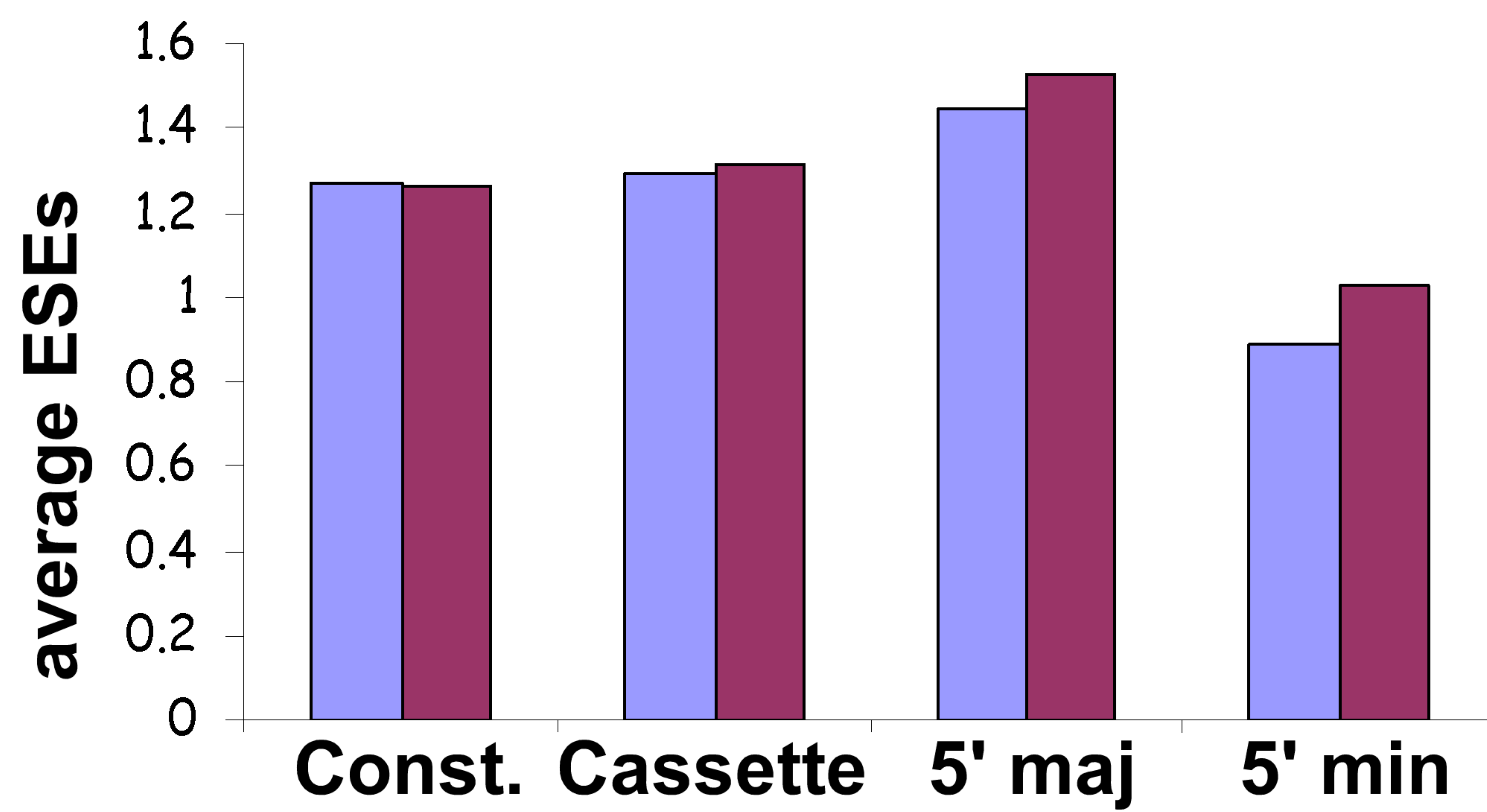**C**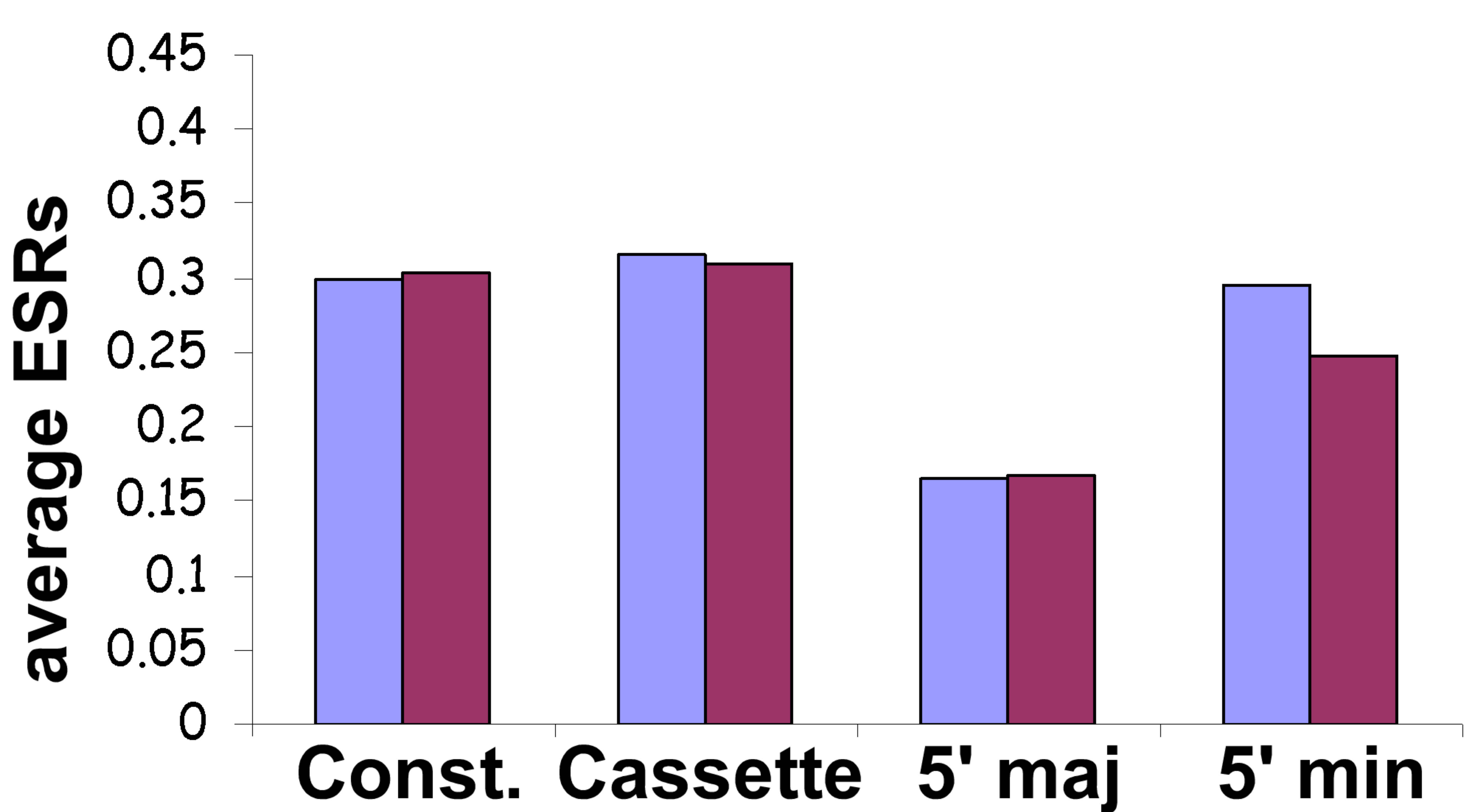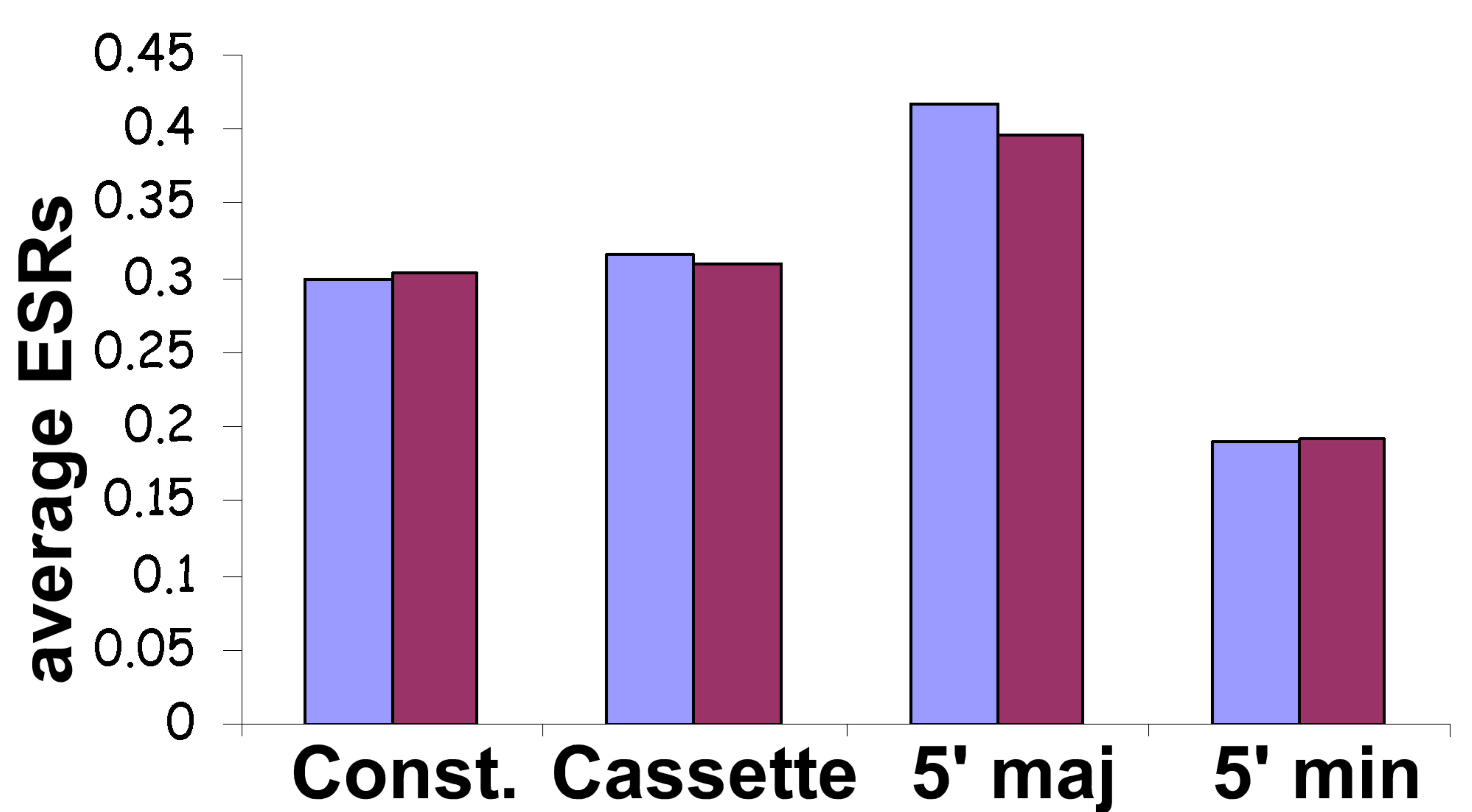**D**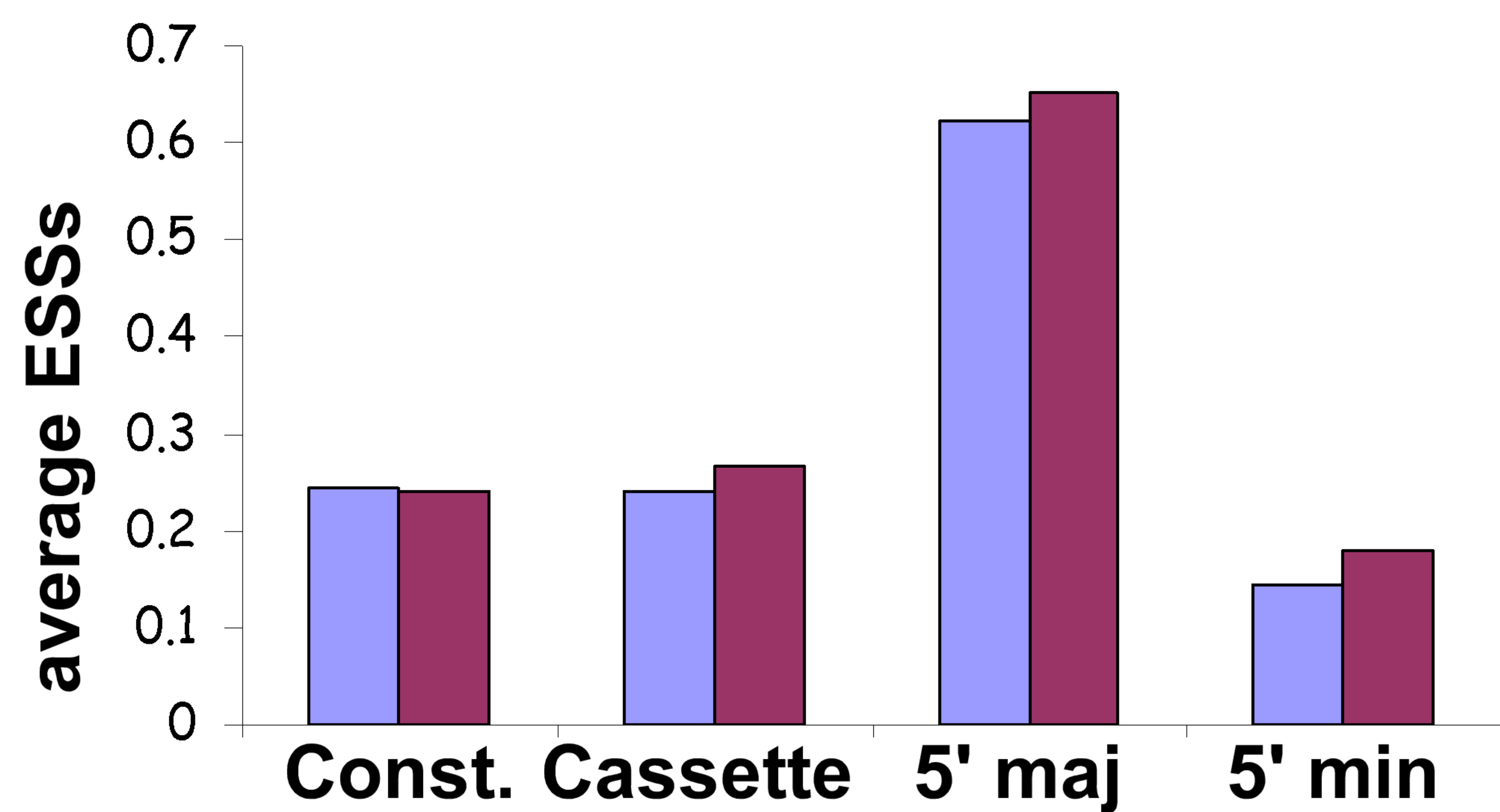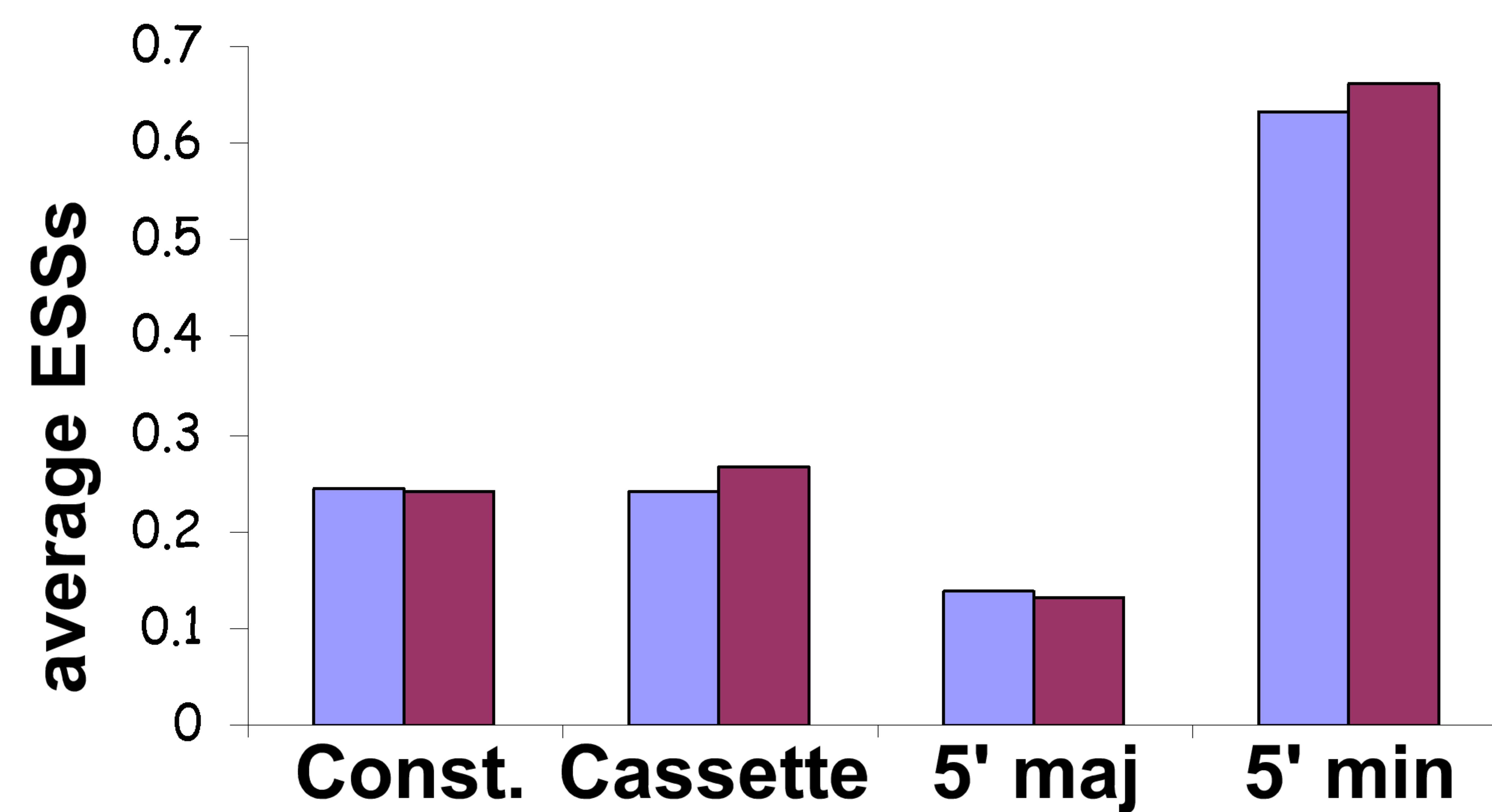**Figure S2**

Supplement: Figure S2 — A region 15 bp upstream of the major (5′ maj) and minor (5′ min) 5′ss was screened for ESRs. Average ESR number was calculated for each region. The analysis was done for human–mouse conserved 5′ alternative splicing events that were divided into two subgroups according to their major/minor forms (see Materials and Methods) and compared to constitutive (Const.) and alternative cassette conserved exons. Left and right panels are “group 2” (major form is longer than minor form) and “group 1” (major form is shorter than minor form) A5Es, respectively. Human and mouse results are in light blue and purple, respectively. (A) Schematic illustration of the analysis conducted. (B) Average ESEs [36]. (C) Average ESRs [10]. (D) Average ESSs [37]. (323 KB PDF) [file pcbi.0030095.sg002.pdf]

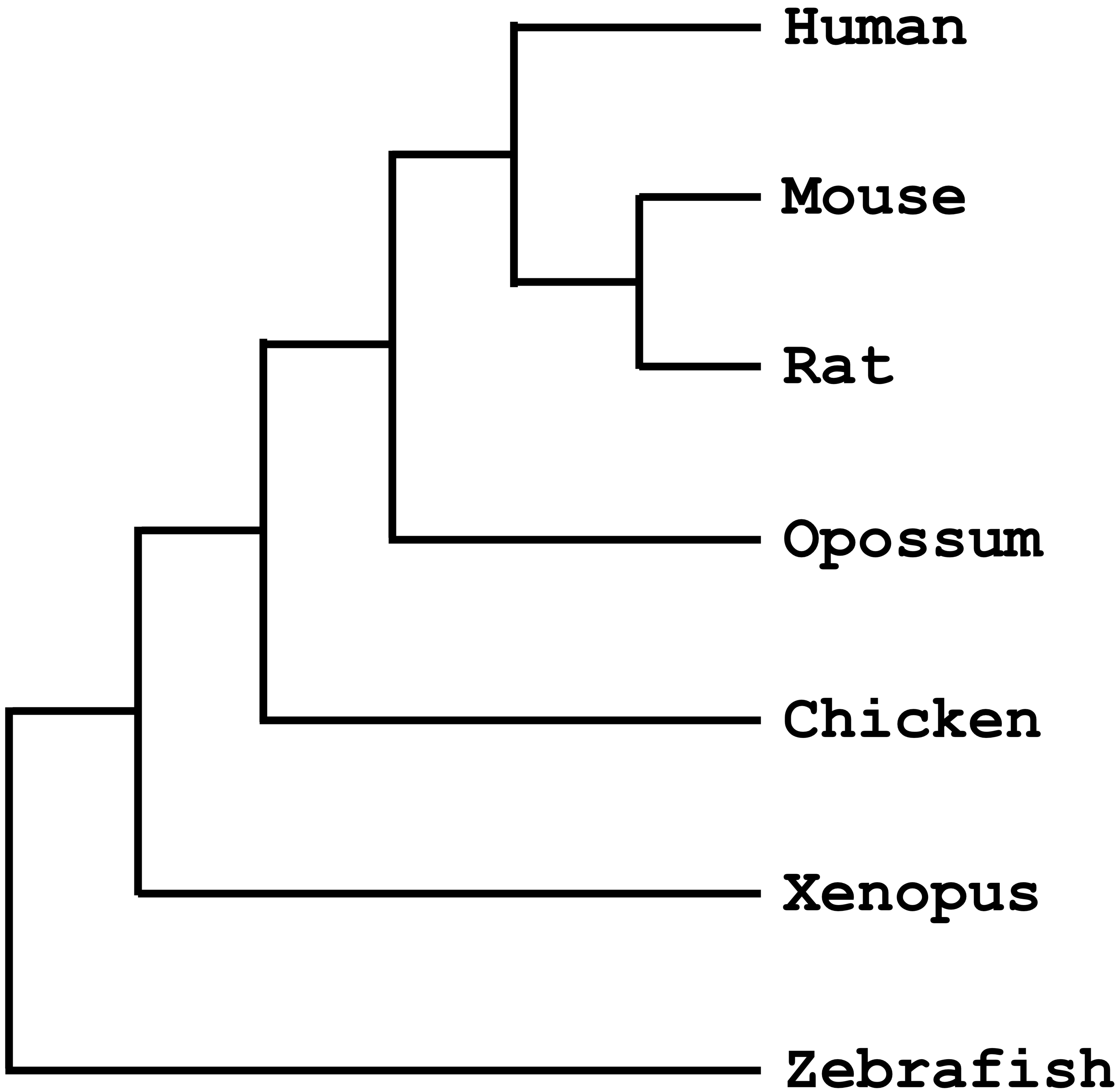

**Figure S3**

Supplement: Figure S3 — A phylogenetic evolutionary tree spanning the seven analyzed species built based on previous evolutionary studies [51]. (102 KB PDF) [file pcbi.0030095.sg003.pdf]

**A****upstream intron**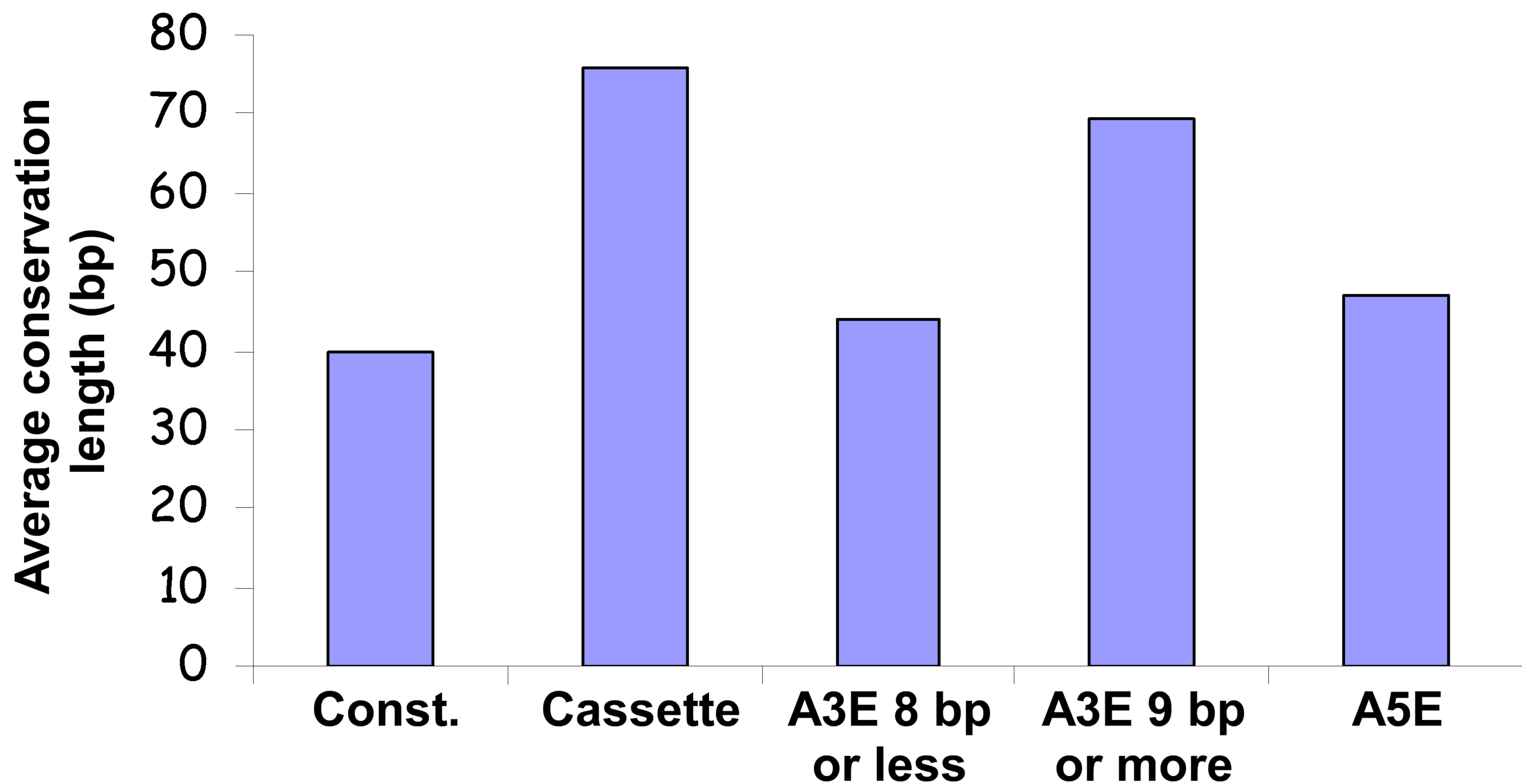**B****downstream intron**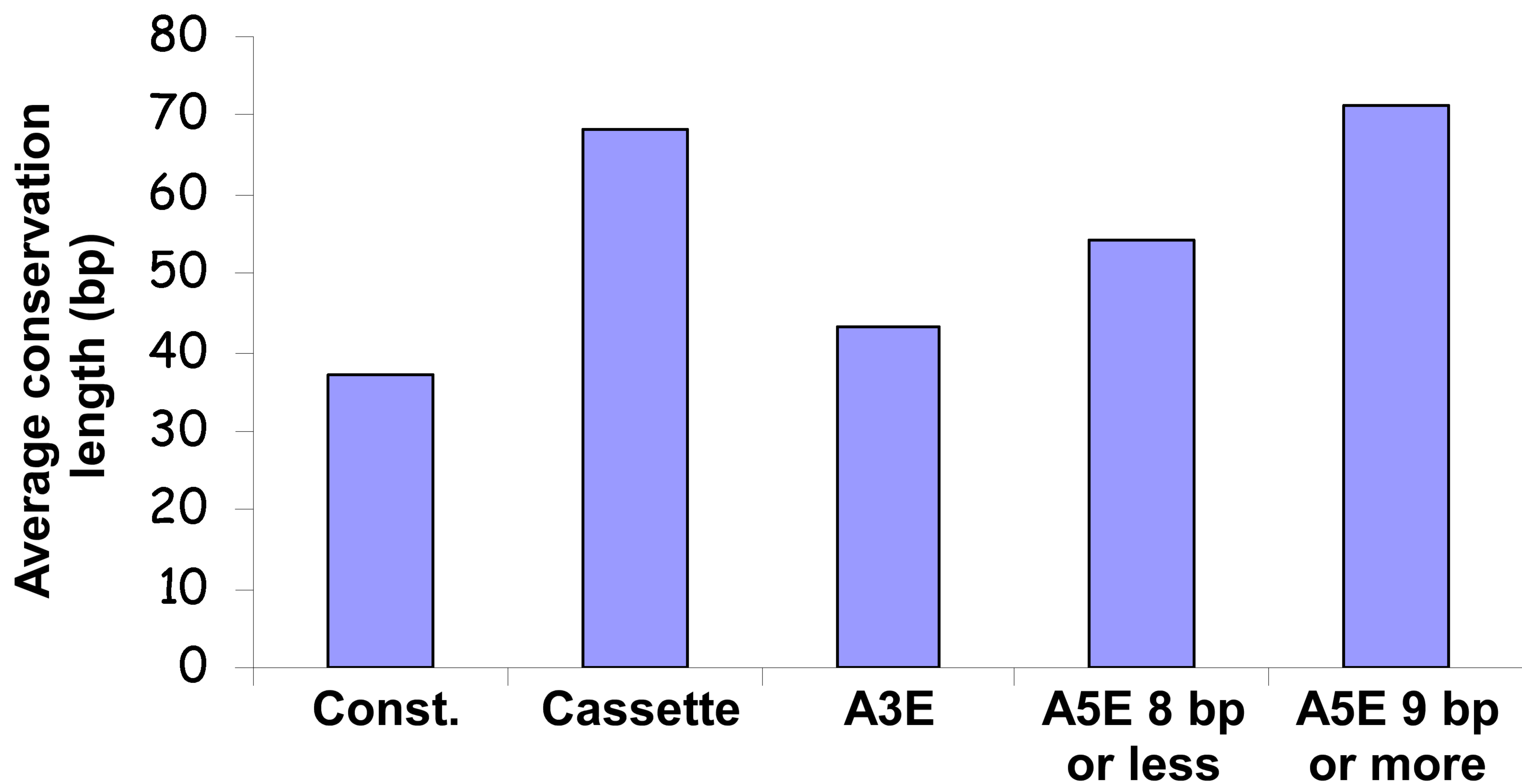**Figure S4**

Supplement: Figure S4 — Alignment of flanking intron regions was performed using the local alignment program Sim4 for upstream and downstream flanking intron (A and B, respectively). The x-axis represents the group of exons and the y-axis represents the average conservation length (bp). (180 KB PDF) [file pcbi.0030095.sg004.pdf]
