# Supplementary material for: The Emergence of Alternative 3′ and 5′ Splice Site Exons from Constitutive Exons
Source: PLoS Comput Biol. 2007 May 25;3(5):e95. doi: 10.1371/journal.pcbi.0030095 (PMC1876488; doi:10.1371/journal.pcbi.0030095)
Supplement: Table S1 — (39 KB DOC) [file pcbi.0030095.st001.doc]

**Table S1:** **Accession numbers of the seven analyzed vertebrate species**

A

|  | **Human** | **Mouse** | **Rat** | **Opossum** | **Chicken** | **Xenopus** | **Zebrafish** |
| --- | --- | --- | --- | --- | --- | --- | --- |
| **Group 1** | NM_004698 | BC026607 | CB586372 | Gene Prediction | NM_001031390 | CX437471 | CK028262 |
| **Group 2** | NM_020131 | NM_033526 | CB747083 | Gene Prediction | NM_001031373 | NM_001037720 | NM_213356 |

B

|  | **Human** | **Mouse** | **Rat** | **Opossum** | **Chicken** | **Xenopus** | **Zebrafish** |
| --- | --- | --- | --- | --- | --- | --- | --- |
| **Group 1** | NM_022496 | CK617284 | CK365697 | Gene Prediction | AJ719762 | NM_001016472 | BC045961 |
| **Group 2** | NM_006311 | AB093281 | CB546074 | Gene Prediction | BU326580 | BC099620 | CK015548 |

Accession numbers are given for A3Es and A5Es (A and B, respectively).
