# Supplementary material for: The Emergence of Alternative 3′ and 5′ Splice Site Exons from Constitutive Exons
Source: PLoS Comput Biol. 2007 May 25;3(5):e95. doi: 10.1371/journal.pcbi.0030095 (PMC1876488; doi:10.1371/journal.pcbi.0030095)
Supplement: Table S2 — (58 KB DOC) [file pcbi.0030095.st002.doc]

**Table S2: Primers used in the experimental validation**

| **sequence** | **Name of primer** |
| --- | --- |
| GATCGAGCCTCGGTCAAGGAG | F(H-UBQLN4) |
| GCATCTGAAGAGGCACTGCCAG | R(H-UBQLN4) |
| GAGACGAGGCCCCAGATTCG | F(M-UBQLN4) |
| CTGCGATCAAGCCTCGGTCAAGGAG | R(M-UBQLN4) |
| GATCAAGCCTCGGTCAAGGA | F(R-UBQLN4) |
| GCATCTGAAGTAGTATTGCCAG | R(R-UBQLN4) |
| CAAAGAAGAGATTTCCAGGCG | F(C-UBQLN4) |
| GCTGGATGTGGAAGGCTGAGAT | R(C-UBQLN4) |
| CCGAAGGATAAAGAAGAAATAG | F(Z-UBQLN4) |
| AGTATATTGGCTGGCTGTGATG | R(Z-UBQLN4) |
| GAGAATTTCTAATTTGATGCGAG | F(H-PRPF3) |
| CACGTTGACATCCTTGTGCAG | R(H-PRPF3) |
| GAGAATCTCAAATTTAATGCGAG | F(M-PRPF3) |
| CACGTTGACATCTTTGTGCAG | R(M-PRPF3) |
| GAGGATCTCCAATTTAATGCGAG | F(R-PRPF3) |
| CACGTTGACATCCTTGTGCAG | R(R-PRPF3) |
| AAGAATTTCAAACTTGATGCGAG | F(C-PRPF3) |
| CCACGTTGACATCTTTGTGTAG | R(C-PRPF3) |
| GAGGATATCAAATCTCATGCGAG | F(X-PRPF3) |
| CACATTGACATCTTTATGTAACAC | R(X-PRPF3) |
| GCGGATCTCCAATCTGATGAG | F(Z-PRPF3) |
| CGATGTTGACATCTTTATGCAGTAC | R(Z-PRPF3) |
| CTCAGAACAGCCTGGCCGA | F(H-NCOR1) |
| CCTCTGGCTGATAGGTCTGCAG | R(H-NCOR1) |
| CAGAACAGCCAGGCCGTCC | F(M-NCOR1) |
| TCTGGCTGATAGGCCTGTGG | R(M/R-NCOR1) |
| GTTCAGAACAGCCAGGCCG | F(R-NCOR1) |
| GCAGCTCACTCCTGGAGCTC | F(C-NCOR1) |
| CTCTTGTTGATAGGGCTGTAGC | R(C-NCOR1) |
| GAGCAGCCTGTTCGACCAG | F(Z-NCOR1) |
| GATGGGAAGGATCCTTCGCTC | R(Z-NCOR1) |
| CTAATTGTCAGTTCCGGTC | F(H-ACTR6) |
| CATTTACTCTTAATACTGCTTG | R(H/M/R-ACTR6) |
| CCAATTGTCAGTTCAGATC | F(M/R-ACTR6) |
| CAACTGCCAGTTCCGATC | F(C-ACTR6) |
| CATTTACTCTAAGAACTGCTTGAA | R(C-ACTR6) |
| CCAACTGTCAGTTCAGAAC | F(X-ACTR6) |
| CATTAATTCTGAGAGCTGCCTG | R(X-ACTR6) |
| CCAAATAGCCAGTTCCGCAC | F(Z-ACTR6) |
| CGTTAATTCTGAGCGCTGACTG | R(Z-ACTR6) |
